# Structural Revisions in Natural Ellagitannins

**DOI:** 10.3390/molecules23081901

**Published:** 2018-07-30

**Authors:** Hidetoshi Yamada, Shinnosuke Wakamori, Tsukasa Hirokane, Kazutada Ikeuchi, Shintaro Matsumoto

**Affiliations:** 1 School of Science and Technology, Kwansei Gakuin University, Sanda 669-1337, Japan; shinnosuke1010@kwansei.ac.jp (S.W.); ean89778@kwansei.ac.jp (S.M.); 2 Faculty of Pharmaceutical Sciences, Tokushima Bunri University, Tokushima 770-8514, Japan; hiro-tks@ph.bunri-u.ac.jp; 3 Department of Chemistry, Faculty of Science, Hokkaido University, Sapporo 060-0810, Japan; ikeuchi@sci.hokudai.ac.jp

**Keywords:** ellagitannin, structure, revision

## Abstract

Ellagitannins are literally a class of tannins. Triggered by the oxidation of the phenolic parts on β-pentagalloyl-d-glucose, ellagitannins are generated through various structural conversions, such as the coupling of the phenolic parts, oxidation to highly complex structures, and the formation of dimer and lager analogs, which expand the structural diversity. To date, more than 1000 natural ellagitannins have been identified. Since these phenolic compounds exhibit a variety of biological activities, ellagitannins have potential applications in medicine and health enhancement. Within the context of identifying suitable applications, considerations need to be based on correct structural features. This review describes the structural revisions of 32 natural ellagitannins, namely alnusiin; alnusnin A and B; castalagin; castalin; casuarinin; cercidinin A and B; chebulagic acid; chebulinic acid; corilagin; geraniin; isoterchebin; nobotanin B, C, E, G, H, I, J, and K; punicalagin; punicalin; punigluconin; roxbin B; sanguiin H-2, H-3, and H-6; stachyurin; terchebin; vescalagin; and vescalin. The major focus is on the outline of the initial structural determination, on the processes to find the errors in the structure, and on the methods for the revision of the structure.

## 1. Introduction

Tannins are astringent polyphenolic compounds with high diversity in biological activities [1,2,3]. Many of the compounds have antioxidative effects and affect various organisms, such as fungi, tumor cells, and viruses. Originally, the term tannin meant a compound that could be used for the tanning of animal hides. Today, tannins are divided into multiple classes according to the source and the chemical structures; however, they are roughly classified into two categories: condensed and hydrolyzable tannins [4,5]. Of the two, ellagitannins belong to the hydrolyzable tannins.

**Figure 1:**
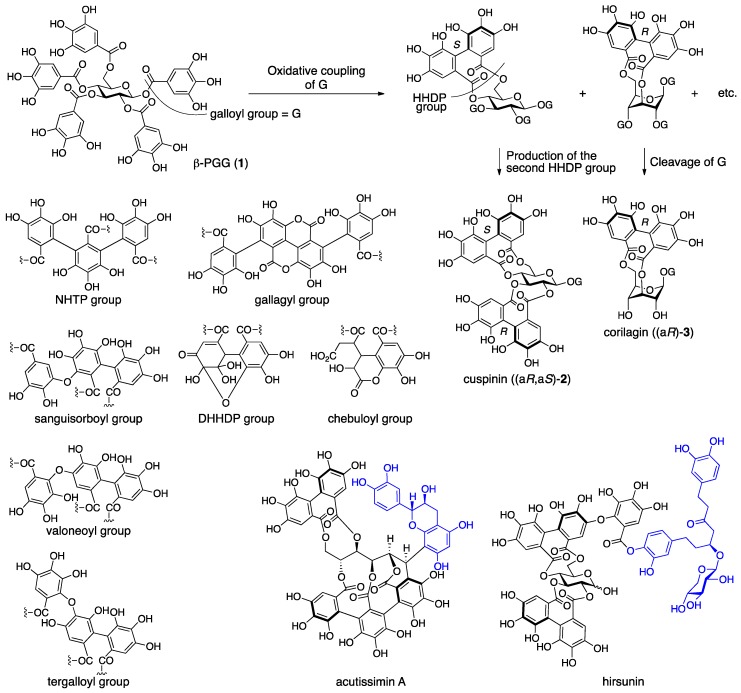
The oxidative intramolecular coupling of the galloyl groups and the structures of components of ellagitannin, acutissimin A, and hirsunin.

Ellagitannins arise through biosynthetic pathways that allow for the production of diversity. In the early stage of the biosynthesis, the galloyl groups of the β-pentagalloyl glucose (**1**) couple oxidatively to produce the hexahydroxydiphenoyl (HHDP) group (Figure 1) [6,7,8,9,10]. Two of the five galloyl groups of **1** can couple and, in addition, the occurrence of *R* or *S* axial chirality on the HHDP group may be possible. Therefore, the number of combinations is 20 (_5_*C*_2_ × 2). After the occurrence of the first HHDP group, the production of the second HHDP group as cuspinin ((a*R*,a*S*)-**2**) and the cleavage of the ester bonds of the HHDP and the rest of the galloyl groups as corilagin ((a*R*)-**3**) may expand the variation. The oxidative coupling continues further to construct the trimer and tetramer of the galloyl group, which are called the nonahydroxytriphenoyl (NHTP) (alias: flavogallonyl) and gallagyl groups, respectively [11,12]. In addition, the galloyl and HHDP groups can connect oxidatively through a C–O bond, which produces sanguisorboyl, tergalloyl, valoneoyl, and other groups [13]. Production of the C–O connected components often involves the generation of dimeric and larger ellagitannins to drastically increase the diversity. The HHDP group can be further oxidized to form the dehydrohexahydroxydiphenoyl (DHHDP), chebuloyl, and other groups, which brings the structures beyond the patterned restriction. Further, ellagitannins can join to the components outside of ellagitannins, such as acutissimin A and hirsunin, to illustrate illimitable possibilities in structure [14,15].

The structural diversity expands in the direction that makes the structures become more complex. Today, structures of complicated ellagitannins can be elucidated. However, during their development, studies for structural determinations had tackled the most difficult subjects at the time. Therefore, situations in which it was difficult to narrow down the options or which led to incorrect structures could not be avoided. The reported structures have been exposed to verification by history, including the development of analytical methods and confirmation by chemical synthesis, and some of them were revised. In the meantime, the reported structures were diffused by a secondary medium, such as books, reviews, and websites. Although the structures were revised afterward, most of the exhibited information remains as it was, which causes confusion and misunderstanding. Hence, reviewing structural revisions is worthwhile as Amagata and McPhail have done already in an independent publication [16,17]. However, there has been no review article featuring structural revisions of ellagitannins, which is the topic of this article.

## 2. Notice

**Figure 2:**
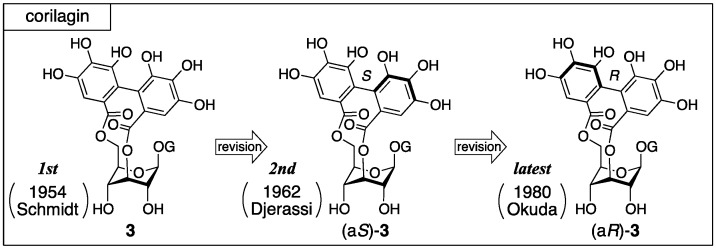
The transition of the structure of corilagin.

In this review article, the following contrivance is used in order to improve intelligibility. For the revised compounds, all of the previously reported structures are exhibited inside a square frame with angular corners (for example, Figure 2). In the frame, the words “*1st*”, “*2nd*”, “*3rd*”, and “*latest*” indicate the transition of the structure. As a guide, the reported year and the representative of the report are appended in a parenthesis. Arrows with “revision” also show the transition. The flame appears first in each description for revised ellagitannins, which might help in understanding the overall transition. Regarding the initial structural determination, the processes to find the errors in the structure, and the methods for the revision of the structure, glancing at figures might not be enough to understand; so, reading the text while referring to the figures is recommended. Degraded compounds that arose in the processes of the structural determination are also illustrated similarly in square frames with angular corners (for example, Figure 10). Frames with round corners are used to indicate the simplified expression of frequent components (for example, Figure 8) and to separate adjacent figures clarifying different contents (for example, Figure 10). We refer to more than 100 original papers in this review article. Except for References [18,19], every outcome was published by several authors. However, when we refer to authors in the review, we restricted ourselves to a delegate to avoid the repeating phrase “and co-workers”, of course, with respect for all of the co-workers.

## 3. Structural Revision in Ellagitannins

### 3.1. Correction of the Bonding Positions of the Galloyl and HHDP Groups and Correction of the Axial Chirality of the HHDP Group

#### 3.1.1. Corilagin

Corilagin was first isolated from dividivi (*Caesalpinia coriaria*) by Schmidt in 1951 [20]. Three years later, the first structure (**3**) was determined (Figure 2), where the axial chirality of the HHDP group remained unknown [21]. In the second structure, the axial chirality was revealed as *S* by Djerassi on the basis of an empirical rule named the amide rule [22]. After that, Okuda revised the axial chirality to be *R* with undeniable facts [23].

**Figure 3:**
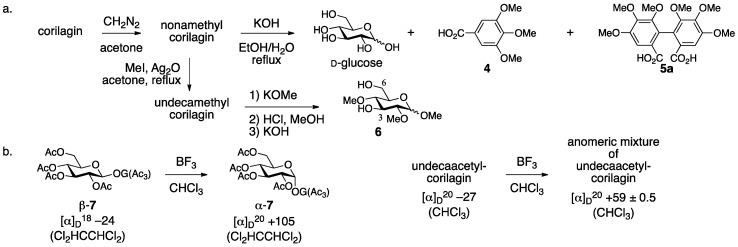
(**a**) The successive methylation and degradation of corilagin and (**b**) the basis for β-stereochemistry at the anomeric position.

The first structure **3** was determined by methylation/degradation and comparison of the fragments with known analogs [21]. The treatment of corilagin with diazomethane provided nonamethylcorilagin, the hydrolysis of which released d-glucose, tri-*O*-methylgallic acid (**4**), and hexamethoxydiphenic acid (**5a**) (Figure 3a) [24]. Therefore, corilagin is an esterified glucose with galloyl and HHDP groups. On the other hand, the methylation of nonamethylcorilagin with MeI/Ag_2_O yielded undecamethylcorilagin, the methanolysis of which produced a mixture of an anomeric isomer that arose by the release of the galloyl moiety. The subsequent transformation of the hemiacetal to the corresponding methyl acetal followed by the hydrolysis of the remaining HHDP group furnished 1,2,4-tri-*O*-methylglucose (**6**) [25,26]. These results were evidence for the structure **3** that possessed the HHDP group bridging between the O-3 and O-6 of glucose and the galloyl group at O-1. The β-stereochemistry came from the behavior of a specific optical rotation. Thus, according to the knowledge that the anomeric isomerization of levorotatory β-**7** provides dextrorotatory α-**7** (Figure 3b) [27], levorotatory undecaacetylcorilagin provided an anomeric mixture of undecaacetylcorilagin after a similar isomerization whose specific optical rotation in gross was dextrorotatory. Later on, Schmidt confirmed the structure **3** using a ^1^H-NMR spectrum [28].

**Figure 4:**
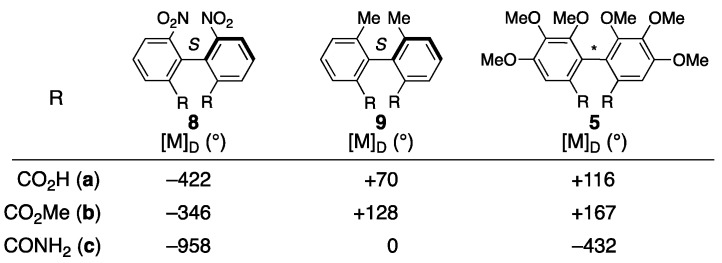
The data used for the determination of the *S*-axial chirality in the second structure of corilagin.

The axial chirality in (a*S*)-**3** was attributed to an empirical rule for the prediction of axial chirality employing optical rotatory dispersion (ORD) spectra [22]. The grounds for the rule are the behavior of the ORD values of the amides **8c** and **9c**, which are smaller than those of the respective carboxylic acids **8a** and **9a** and methyl esters **8b** and **9b** (Figure 4). Djerassi compared the ORD values of the dicarboxylic acid derivative of the HHDP group **5a**, the methyl ester **5b**, and the amide **5c** to find that the value of **5c** was obviously smaller than those of **5a** and **5b**; here, **5b** and **5c** were derived from the carboxylic acid (+)-**5a** [29]. According to the observation, the axial chirality of the HHDP group was determined to be *S* [22].

**Figure 5:**
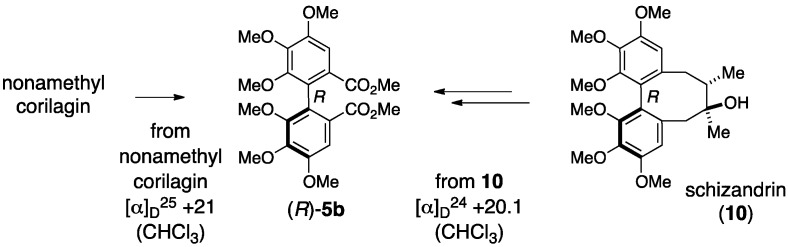
The determination of the *R*-axial chirality in the latest structure of corilagin.

Okuda corrected the axial chirality of corilagin to give the latest structure (a*R*)-**3** in a reliable manner. They confirmed that both of the specific optical rotations were dextrorotatory between the two dimethyl esters **5b** derived from nonamethylcorilagin and from the schizandrin (**10**) bearing the definite *R*-axial chirality; hence, the axial chirality in corilagin was *R* (Figure 5) [23]. After that, Okuda and Seikel independently clarified that the conformation of the glucopyranose core was in ^1^*C*_4_ by ^1^H-NMR analyses [30,31]. In addition, corilagin was obtained as a hydrolysate of geraniin (Section 3.2.1) [32], the structure of which was elucidated by an X-ray diffraction study of the singe crystal [33]. Afterward, Yamada synthesized (a*R*)-**3** [34].

#### 3.1.2. Punigluconin

**Figure 6:**
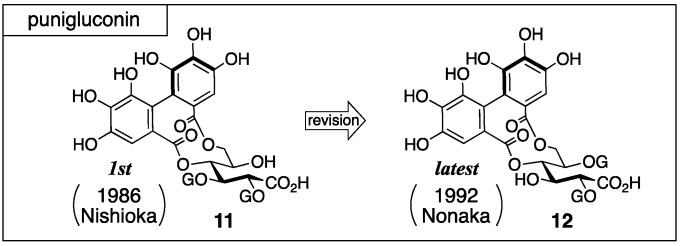
The transition of the structure of punigluconin.

Punigluconin was isolated from *Punica granatum* L. by Nishioka [35]. For the first structure, **11** that possessed the galloyl groups on O-2 and O-3 of gluconic acid was reported (Figure 6). After that, the structure was revised to the 2,5-di-*O*-galloylated **12**, which is the latest structure [36].

**Figure 7:**
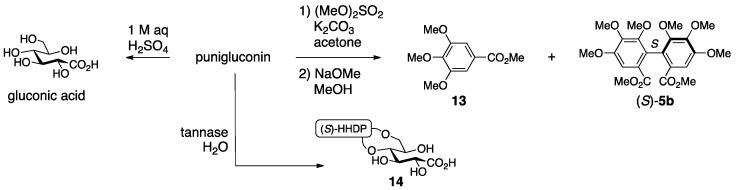
The transformations used in the determination for the first structure of punigluconin (**11**).

The structure **11** was determined on the basis of an NMR study and chemical transformations [35]. ^1^H-NMR indicated that punigluconin possessed two galloyl groups and one HHDP group. ^13^C-NMR exhibited the existence of an aldonic acid. The treatment of punigluconin with diluted sulfuric acid produced gluconic acid (Figure 7); hence, the aldonic acid was gluconic acid. On the other hand, the full methylation of punigluconin followed by methanolysis provided methyl tri-*O*-methylgallate (**13**) and the dimethyl ester (*S*)-**5b** [37] derived from the (*S*)-HHDP group. The ^1^H-NMR spectrum of the tannase-hydrolysate of punigluconin showed a high-field shift of H-2 and H-3. No remarkable change in the chemical shifts was observed on H-4 and H-6. Thus, the hydrolysate was **14**.

**Figure 8:**
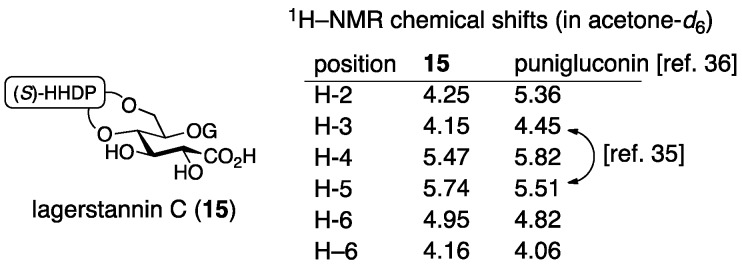
The comparison of the ^1^H-NMR chemical shifts between lagerstannin C (**15**) and punigluconin.

After that, Nonaka isolated lagerstannin C from *Lagerstroemia speciose* (L.) PERS. and determined its structure to be **15** (Figure 8) [36]. Comparison of the ^1^H-NMR spectrum of punigluconin to that of **15** showed a notable difference at H-2 and similar values at the other hydrogens. Accordingly, the position of the galloyl group was changed to revise the structure to be **12**. Note that the ^1^H-NMR assignments of H-3 and H-5 in the reports of the structural determinations for **11** [35] and for **12** [36] were swapped, which seemed to cause the error in the initial structure.

#### 3.1.3. Cercidinin A and B

**Figure 9:**
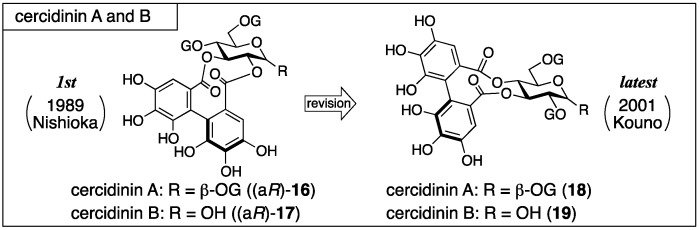
The transition of the structure of cercidinin A and B.

Cercidinin A and B were isolated from *Cercidiphyllum japonicum* SIEB. *Et* ZUCC by Nishioka and first given the structures (a*R*)-**16** and (a*R*)-**17** (Figure 9), respectively [38]. Khanbabaee synthesized (a*R*)-**16** and (a*R*)-**17** in 1998, but the synthesized compounds were different from natural cercidinin A and B [39]. Following the result, Kouno revised the structure to **18** [40]. Yamada synthesized **18** to confirm the structure in 2013 [41].

**Figure 10:**
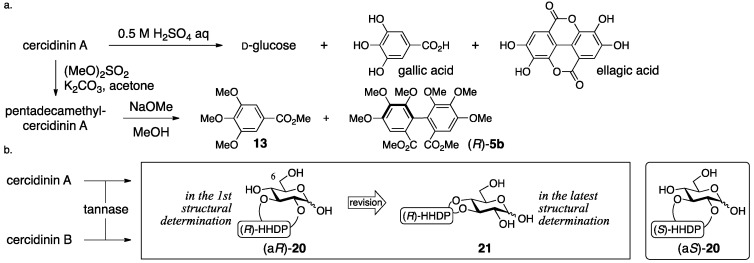
The transformations used in the determination for the first structure of cercidinin A ((a*R*)-**16**) and B ((a*R*)-**17**).

The process for the determination of (a*R*)-**16** and (a*R*)-**17** consists of NMR studies and the degradation of the natural products. The ^1^H-NMR spectrum of cercidinin A indicated that the compound consisted of three galloyl groups and one HHDP group and that all the hydroxy groups of the glucose moiety were acylated. The hydrolysis of cercidinin A produced d-glucose, gallic acid, and ellagic acid (Figure 10a). On the other hand, the methanolysis of pentadecamethylcercidinin A yielded methyl tri-*O*-methylgallate (**13**) and dimethyl hexamethoxydiphenoate ((*R*)-**5b**). The *R*-axial chirality was based on the comparison of the specific optical rotation of **5b** to the data in the literature [42]. In the ^1^H-NMR data of pentadecamethylcercidinin A, the coupling constants of the hydrogens on glucose displayed that the conformation of the glucose part was in the typical ^4^*C*_1_ form. The tannase-hydrolysate of cercidinin A was a mixture of anomers (Figure 10b). In addition, the chemical shift of H-6 of the tannase-hydrolysate shifted to a higher magnetic field than that of cercidinin A. Therefore, the HHDP group was situated at the 2,3- or 3,4-positions of the glucose moiety. The reason for the decision on the 2,3-HHDP isomer (a*R*)-**20** was the similarity of the ^1^H-NMR spectra (100 MHz) to that of the known (a*S*)-**20** [43]. The β-anomeric stereochemistry in cercidinin A was supported by the coupling constant of H-1 (*J* = 8 Hz) in the ^1^H-NMR spectrum. The ^1^H-NMR of cercidinin B exhibited that the compound possessed two galloyl groups and one HHDP group and that a hydroxy group was situated at the anomeric position. The hydrolysis of cercidinin B using tannase provided the same compound with the tannase-hydrolysate of cercidinin A.

**Figure 11:**
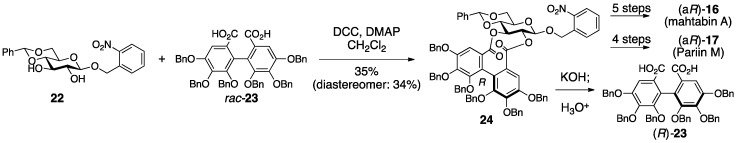
The synthesis of (a*R*)-**16** and (a*R*)-**17**.

In Khanbabaee’s synthesis that indicated the error in (a*R*)-**16** and (a*R*)-**17**, both the setting of the position of the HHDP group and the process that determined the axial chirality are reliable (Figure 11) [39]. They synthesized the bridged compound **24** through the double esterification of the 2,3-diol **22** with the racemic diphenic acid *rac*-**23**. The obtained bislactone was a mixture of diastereomers which was separable by column chromatography. The *R*-axial chirality was ascribed to the dextrorotation of **23**, that is, the hydrolysate of **24**, because its hexamethyl analog (*R*)-**5a** had been known to be dextrorotatory (Figure 4) [42]. The conversion of **24** through several steps produced (a*R*)-**16** and (a*R*)-**17**, which were eventually found to be different from natural cercidinin A and B. Namely, both (a*R*)-**16** and (a*R*)-**17** actually represent new compounds, which were subsequently named mahtabin A and Pariin M, respectively. 

**Figure 12:**
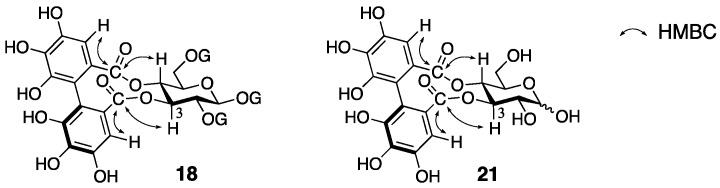
The significant HMBC relationships used for the determination of the structure **18** and **21**.

Nishioka’s structural revisions are grounded on the following observations. Reviewing the logic for the first structure (a*R*)-**16**, the determinations of the axial chirality and of the conformation of the glucose moiety were factually correct. The assignment of all the hydrogens and carbons of the carbohydrate part in cercidinin A was possible by employing a 500 MHz NMR instrument. On the basis of the assignments, the specification of the 3,4-HHDP structure was possible using hetero-nuclear multiple-bond coherence (HMBC) correlations between the carbonyl carbons of the HHDP group and the H-3/H-4 as displayed on the structure of **18** (Figure 12). Furthermore, the HMBC experiment determined the structure of **21** that was the hydrolysate of cercidinins with tannase. These results advocated for the structure **18**. In Nishioka’s report, the structural revision of cercidinin A was only described. However, the structure of cercidinin B should be corrected to **19** as the difference between cercidinin A and B has been known to be the existence or non-existence of the anomeric galloyl group [38].

**Figure 13:**
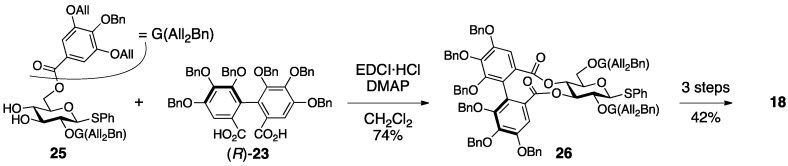
The key steps in the synthesis of **18**.

In Yamada’s synthesis of **18**, a protected (*R*)-HHDP diacid **23** is introduced between the O-3 and O-4 of glucose (Figure 13) [41]. The double esterification of the diol **25** with the dicarboxylic acid (*R*)-**23**, the enantiomeric purity of which was 100%, yielded the bislactone **26**. The subsequent three steps provided **18**, which was identical to natural cercidinin A.

#### 3.1.4. Roxbin B

**Figure 14:**
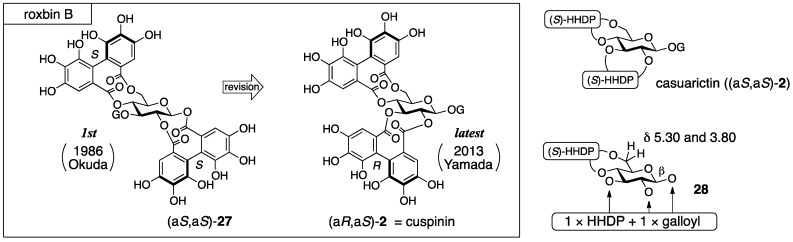
The transition of the structure of roxbin B, the structure of casuarictin ((a*S*,a*S*)-**2**), and the reliable partial structure **28** of roxbin B.

Roxbin B was isolated from *Rosa roxburghii* TRATT and first given the structure (a*S*,a*S*)-**27** by Okuda (Figure 14) [44]. After that, Yamada synthesized (a*S*,a*S*)-**27**, but the synthesized compound was not identical to the natural product [45]. Yamada reviewed the process of the structural determination for (a*S*,a*S*)-**27**, presumed on the basis of the review that roxbin B was the same compound with cuspinin ((a*R*,a*S*)-**2**), and confirmed the presumption by total syntheses [46].

The structure (a*S*,a*S*)-**27** was determined by degradation, ^1^H-NMR and circular dichroism (CD) spectra, and an HPLC chromatogram [44]. The ^1^H-NMR spectrum of roxbin B revealed the numbers of the galloyl and HHDP groups. The hydrolysis of roxbin B produced gallic acid, ellagic acid, and d-glucose. The determination of the *S*,*S*-axial chiralities of the two HHDP groups was based on the Cotton effects that were positive at 225 nm and negative at 255 nm in the CD spectrum. Because the behavior was similar to that of casuarictin ((a*S*,a*S*)-**2**) [37], Okuda assessed that roxbin B was an isomer of (a*S*,a*S*)-**2**. In the ^1^H-NMR spectrum, one of the two H-6s shifted to a lower field (δ5.30 and 3.80, see **28**), which indicated that one of the HHDP groups bridged between O-4 and O-6 [47]. The spectrum also demonstrated the β-anomeric stereochemistry (^3^*J*_H-1–H-2_ = 8.5 Hz) and the ^4^*C*_1_ conformation of the pyranose ring. When the second HHDP group bridged between O-1 and O-3, the conformation of the pyranose should be in a boat form; thus, this could be excluded. The HPLC chromatogram of tannase-treated roxbin B indicated peaks due to gallic acid and another component. If roxbin B was an O-1 gallate, the component would be a mixture of anomers. The authors considered that such a mixture of anomers could be separated into two peaks in the chromatogram; hence, O-1 was judged not to be galloylated. Therefore, they concluded the 1,2-*O*-HHDP structure (a*S*,a*S*)-**27**.

**Figure 15:**
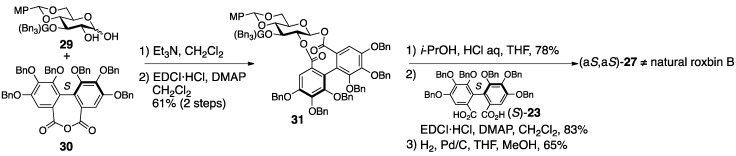
The synthesis of (a*S*,a*S*)-**27**.

In the synthesis of (a*S*,a*S*)-**27**, the introduced position and axial chirality of the HHDP groups were secure [45]. The stepwise esterification of the 1,2-diol **29** and the (*S*)-HHDP acid anhydride **30** (Figure 15), of which the optical purity was >99%, provided the 1,2-*O*-(*S*)-HHDP bridged compound **31**. The removal of the *p*-methoxybenzylidene acetal from **31** released the corresponding 4,6-diol, to which dicarboxylic acid (*S*)-**23** was introduced in a double esterification manner. Finally, the removal of all benzyl groups provided (a*S*,a*S*)-**27**. However, the ^1^H/^13^C-NMR spectra of the synthesized (a*S,aS*)-**27** were totally different from those of the natural roxbin B.

**Figure 16:**
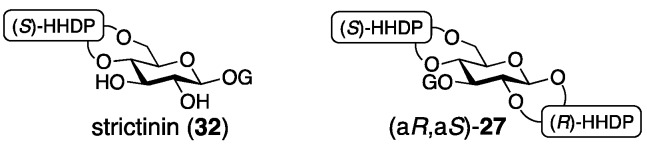
The structure of strictinin (**32**) and of a candidate (a*R*,a*S*)-**27** for revised roxbin B.

The revised structure of roxbin B ((a*R*,a*S*)-**2**) was obtained by reviewing Okuda’s process for the structure (a*S*,a*S*)-**27** [46]. Tracing Okuda’s structural determination, it was trustworthy that roxbin B contained the partial structure **28** (Figure 14). In the review, a sample of roxbin B preserved for more than 20 years was found to degrade and provide strictinin (**32**) (Figure 16). This observation was associated with the 4,6-*O*-(*S*)-HHDP structure. On the other hand, the weak Cotton effect at 236 nm in the CD spectrum raised a doubt about whether the axial chiralities of the HHDP groups were both *S*. Thus, a hypothesis that one of the two HHDP groups had an *R*-axial chirality supposed two possible structures, (a*R*,a*S*)-**2** (Figure 14) and (a*R*,a*S*)-**27** (Figure 16). Of the two, (a*R*,a*S*)-**2** was cuspinin that had already been isolated by Nishioka [38]. The data in the literature on cuspinin were in good agreement with those of roxbin B. In Nishioka’s report [38], the three main subjects of discussion were the structures of cercidinin A and B (Section 3.1.3) and cuspinin. Among the three, the structures of cercidinin A and B were revised and that of cuspinin supported the structural revision of roxbin B.

**Figure 17:**
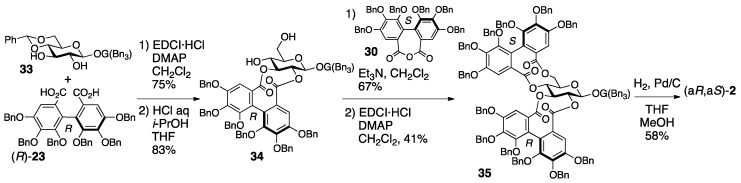
The synthesis of cuspinin ((a*R*,a*S*)-**2**), the structure of which was the revised structure of roxbin B.

The synthesis aiming at confirmation of the structure of cuspinin ((a*R*,a*S*)-**2**), which is the revised structure of roxbin B, is conducted using HHDP compounds with secure axial chirality [46]. The sequential bridge formation between O-2 and O-3 of **33** adopting the HHDP derivative (*R*)-**23**, the removal of the benzylidene acetal, and the introduction of the (*S*)-HHDP group between O-4 and O-6 of **34** provided **35** (Figure 17). The debenzylation of **35** provided (a*R*,a*S*)-**2**, the ^1^H/^13^C-NMR of which were identical to those of cuspinin (and also roxbin B). Moreover, (a*R*,a*S*)-**27** was synthesized similarly and used to illustrate that its ^1^H/^13^C-NMR were obviously different from those of (a*R*,a*S*)-**2**.

**Figure 18:**
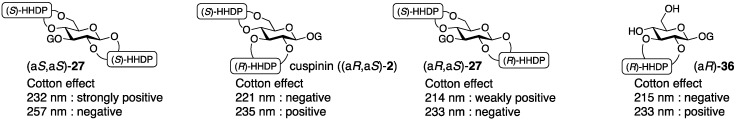
The comparison of the Cotton effects observed on (a*S*,a*S*)-**27**, cuspinin ((a*R*,a*S*)-**2**), (a*R*,a*S*)-**27**, and (a*R*)-**36**.

The supposed cause to reach the first structure (a*S*,a*S*)-**27** was also reported [46]. Thus, from the comparison of the CD spectra of (a*S*,a*S*)-**27** (a synthesized compound), (a*R*,a*S*)-**2**, (a*R*,a*S*)-**27** (a synthesized compound), and (a*R*)-**36** emerged a tendency that the Cotton effect of a compound bearing the (*R*)-HHDP group indicated a 20 nm smaller wavelength than that of an (*S*)-HHDP compound (Figure 18). In addition, in the case where both the (*R*)- and (*S*)-HHDP groups were in a molecule, the intensity of the Cotton effect was weakened due to compensation for each other (compare (a*S*,a*S*)-**27** and (a*R*,a*S*)-**27**, Figure 18). Yamada explained that the determination of the structure (a*S*,a*S*)-**27** was attributed to the unnoticed negative Cotton effect around 220 nm.

### 3.2. Correction Based on the Structure of the DHHDP Group

#### 3.2.1. Geraniin

**Figure 19:**
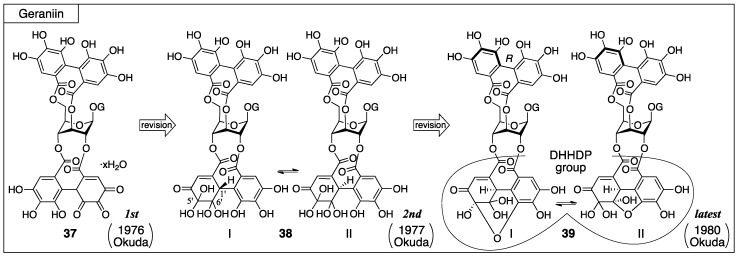
The transition of the structure of geraniin.

For geraniin, its first isolation, the structural determination, and all structural revisions were conducted by Okuda. Geraniin has been isolated from various plants, but initially it was isolated from *Geranium thunbergii* Sieb. *et* Zucc. and **37** was given as the first structure (Figure 19) [32,48]. After that, the structure of the DHHDP group was revised twice. For the second structure, the equilibrium mixture **38** was proposed [49]. Later on, the structure was revised to another equilibrium mixture **39** and the axial chirality of the HHDP group was revealed to be *R* [23]. The hemiacetal structure with the six-membered ring **39**-I was confirmed by an X-ray diffraction study [33].

**Figure 20:**
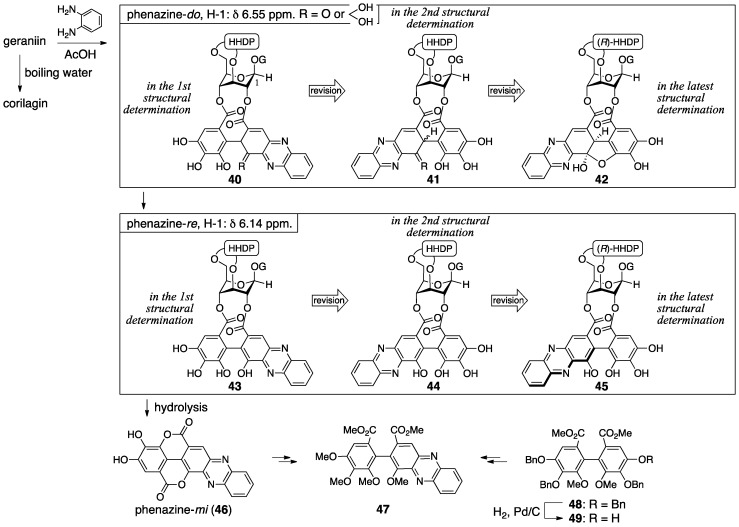
The hydrolysis and derivatization of geraniin.

In the determination of the first structure (**37**), the use of the phenazine derivative is a feature [32]. Boiling water hydrolyzed geraniin to release corilagin (Section 3.1.1) (Figure 20); therefore, geraniin contained corilagin as a partial structure. Because the ^1^H-NMR chemical shift of H-2 and H-4 of geraniin was over 1 ppm larger than those of corilagin, there were esters on O-2 and O-4 in geraniin. The treatment of geraniin with benzene-1,2-diamine and acetic acid produced phenazine-*do*. The phenazine-*do* gradually changed to phenazine-*re*, the hydrolysis of which provided phenazine-*mi* (**46**). The evidence for the structure **46** was the identity of two dimethyl esters **47**, which were derived from **46** and from the known **48** [50] through **49** that was obtained by the hydrogenolytic removal of only one benzyl group from **48** [48]. These results demonstrated that the component on the O-2 and O-4 was a partly oxidized HHDP group, the DHHDP group. In addition, the high-field shift of the H-1 (from 6.55 to 6.14 ppm) in ^1^H-NMR observed in the transformation from phenazine-*do* to -*re* suggested that the phenazine skeleton was on the O-2 side because of the proximity. Hence, the structure of geraniin was **37**. Similarly, the structures of phenazine-*do* and -*re* were decided to be **40** and **43**, respectively. Note that the structure **40** seems to be one that would aromatize immediately, but it was copied as reported.

**Figure 21:**
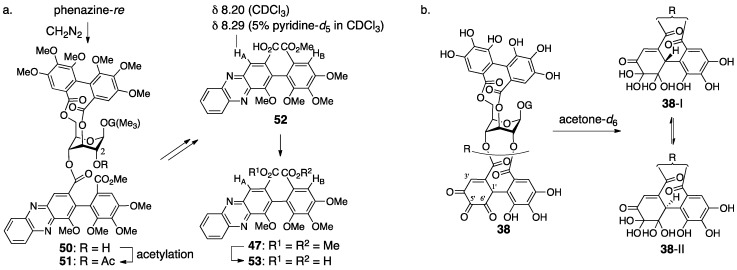
(**a**) The transformations that lead to the second structure of geraniin (**38**) and (**b**) the equilibrium between the two hydrated structures.

The revision to the second structure of geraniin (**38**) was the positional swap of the quinone ring in the 2,4-*O*-DHHDP group [49]. The diazomethane treatment of phenazine-*re* produced **50** (Figure 21a), in which the ester bond “on O-2” was cleaved. The reason for the “on O-2” was the lower field shift of H-2 in the acetylated **51**. In the ^1^H-NMR spectrum of the monocarboxylic acid **52**, which was derived by the hydrolysis of **51**, the chemical shift of H_A_ situated on the phenazine skeleton shifted to the lower field by the addition of pyridine-*d*_5_. Generally, the chemical shift of a neighboring (*o*-position in this case) hydrogen of a carboxylic acid slides to the lower field when the carboxylic acid forms a salt. By contrast, in the ^1^H-NMR spectra of the corresponding dimethyl ester **47** and the dicarboxylic acid **53**, which were derived from **52**, the chemical shifts of H_A_ and H_B_ of **47** showed no significant shifts and both of H_A_ and H_B_ in **53** shifted lower. Thus, the phenazine moiety was on the O-4 side; hence, the structure of geraniin was **38**. This alteration revised the structures of phenazine- *do* and -*re* to be **41** and **44**, respectively (Figure 20). When a crystal of geraniin was dissolved in acetone-*d*_6_ containing 10% D_2_O, the compound was changed to be a 1:1 mixture within 6 h (Figure 21b). On the other hand, more than 12 h was required to reach a 1:1 mixture in anhydrous acetone-*d*_6_. Accordingly, water participated in the equilibrium. The ^1^H-NMR of the equilibrium mixture displayed each set of two signals due to methine hydrogens and vinyl hydrogens. Additionally, in the ^13^C-NMR, each set of the two carbons was observed due to two kinds of hydrated ketones and a carbonyl carbon of conjugated ketone. These results provided the structures **38**-I and -II, which were the epimers at C-1′ and included two hydrated ketones at C-5′ and C-6′.

**Figure 22:**
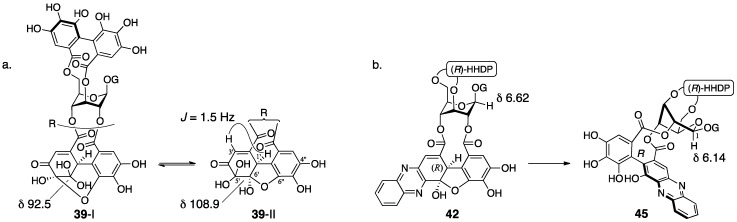
(**a**) The revised hemiacetalic structures and (**b**) the determination of the (*R*)-stereochemistry for C-1′.

The doubt in **38** commenced with the fact that the observation of ^1^H-NMR in the presence of D_2_O did not induce the deuteration of the methine hydrogen [23]. There are two isomers in geraniin, crystalline type-I and non-crystalline type-II that occurs in a solution. Okuda focused on the notable difference of the ^13^C-NMR chemical shifts of C-6′ (Figure 22a) to presume the type-II hemiacetal structure containing the five-membered ring. The structure was associated with the coupling constant between H-1′ and H-3′. The determination of stereochemistries at C-1′ and C-6′ of **39**-II commenced with consideration of the ^1^H-NMR chemical shifts of H-1, which shifted to a higher field when phenazine-*do* was transformed to -*re* (Figure 22b). Okuda inferred that the shielding effect due to the phenazine rings induced the shift and supposed the structure **45**, where the phenazine moiety and H-1 could be close. For the formation of **45** that bears the *R*-axial chirality, the *R*-stereochemistry is necessary for C-1′ in its precursor **42**. The relative configuration between C-1′ and C-6′ was *cis* since **42** had a hemiacetalic structure with a fused five/six-membered ring. Moreover, the precursor **39**-II for **42** included a similar hemiacetalic structure; thus, the stereochemistry of **39**-II was determined to be *1′R*, *6′R*. On the other hand, for the type-I structure, the six-membered ring hemiacetal **39**-I, hydrated **38**-I, and **38**-II were the candidates. Among them, **39**-I was chosen because the ^13^C-NMR spectrum using the deuterium-induced differential isotope shift (DIS) method [51] revealed that the O-6″ was not a hydroxy group. The *R*-axial chirality of the HHDP group in **39** was introduced according to the *R*-chirality in corilagin, which had just been revealed (Section 3.1.1) [23]. In addition, the conformation of the glucopyranose of **39** was determined to be ^1^*C*_4_ in acetone-*d*_6_, which was similar to that in corilagin [30]. Finally, structure **39**-I was confirmed by an X-ray diffraction study [33].

#### 3.2.2. Terchebin

**Figure 23:**
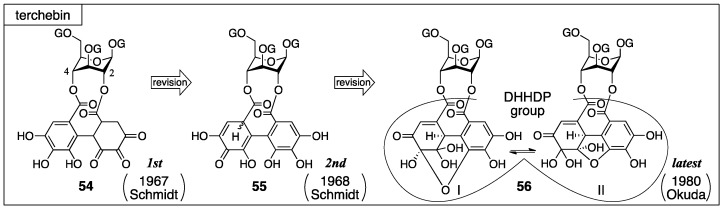
The transition of the structure of terchebin.

Terchebin was isolated by Schmidt from myrobalans (fruit of *Terminalia chebula*) [52]. From this plant, chebulinic acid and chebulagic acid were also isolated (Section 3.3) [52]. Schmidt firstly gave **54** for the structure of terchebin, which possessed a cyclohexane-trione ring on the O-2 side [52]. In the ensuing year, they revised the structure to the mixture of isomers **55** [28]. Later, Okuda further revised the structure to **56** (I and II) that possessed the DHHDP group bridging between O-2 and O-4 (Figure 23) [53].

**Figure 24:**
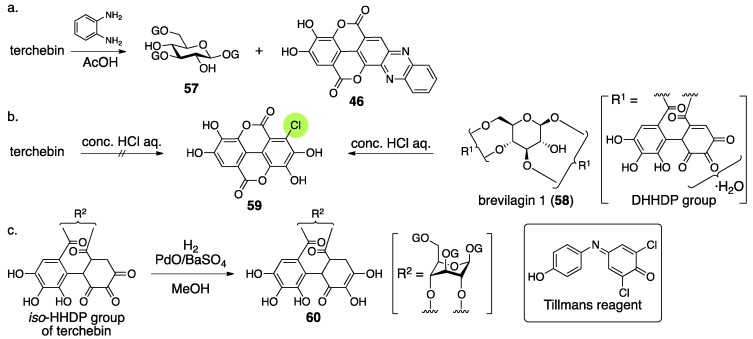
The transformations used for the determination of the first structure of terchebin (**54**).

The structural determination of terchebin began with degradation experiments. The treatment of terchebin with benzene-1,2-diamine and AcOH provided the known compounds 1,3,6-tri-*O*-galloyl-β-glucose (**57**) [54] and phenazine-*mi* **46** (Figure 24a) [55]. According to the observation that **46** was obtained in the structural determination of brevilagin 1 (**58**), they considered that terchebin contained the same DHHDP group bridging between O-2 and O-4 through ester bonds [55]. On the other hand, the treatment of **58** with concentrated hydrochloric acid provided chloroellagic acid (**59**). However, a similar treatment of terchebin did not produce **59**. Furthermore, **58** did not provide a hydrogenated compound but terchebin did (Figure 24c), and the obtained product reduced the Tillmans reagent [56] to suggest that the product contained a reducing structure similar to ascorbic acid. According to these facts, Schmidt reached the conclusion that the structure of the hydrogenated compound was **60** and exhibited the structure of **54**. In the structure, there was no experimental evidence for the direction of the isohexahydroxydiphenoyl (*iso*-HHDP) group. The authors supposed the direction on the basis of the similarity of the first structures of chebulinic acid and chebulagic acid (Section 3.3) [57,58].

**Figure 25:**
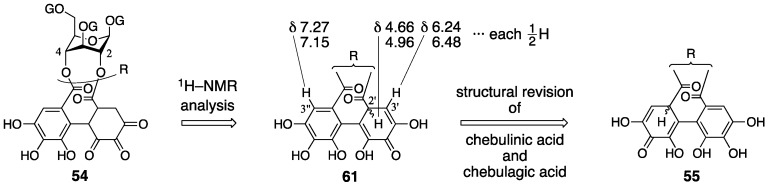
The pathway of the structural revision to **55**.

Consideration of the ^1^H-NMR spectrum of terchebin caused Schmidt to become aware of the error in the structure **54**. The ^1^H-NMR spectrum of terchebin in DMSO-*d*_6_ did not show any signal due to the –CH_2_– group that was in the cyclohexane-trione ring in **54**. Moreover, signals from groups, such as the hydroxy groups other than those of phenol, H-2′, H-3′, and H-3″, were observed as ½ H integrated intensity (Figure 25). According to the observations, Schmidt presumed the diastereomeric mixture **61** for the *iso*-HHDP moiety. With the knowledge of the revisions of the structurally related chebulinic acid and chebulagic acid to their second structures (Section 3.3), structure **55**, in which the benzene ring at the O-4 side was oxidized, was given [28].

**Figure 26:**
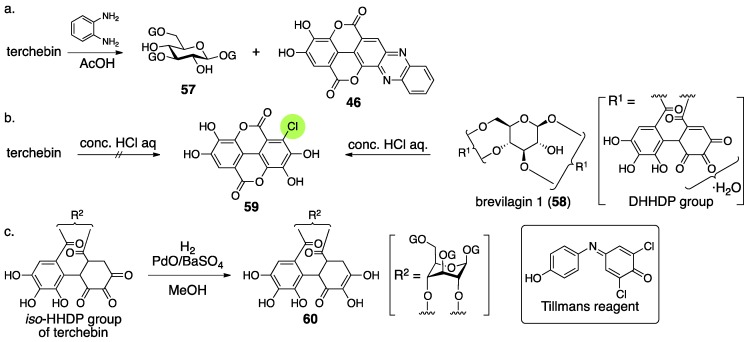
The transformations used for the structural revision to **56**.

According to the structural revision of geraniin (Section 3.2.1) [23], Okuda reinvestigated the structure of terchebin [53]. The hydrolysis of terchebin with concentrated hydrochloric acid followed by methylation provided a mixture, the mass spectrum of which displayed a molecular ion peak that corresponded to tetra-*O*-methylated chloroellagic acid (**62**) (Figure 26). The result was contrary to Schmidt’s report. In addition, Okuda confirmed that the hydrogenated geraniin reduced Tillmans reagent. According to the facts, the existence of the same DHHDP group in geraniin was supposed for the component situated between O-2 and O-4 of terchebin. With the following two observations, Okuda concluded that the structure of terchebin was **56** (I and II). The first observation was that the ^1^H/^13^C-NMR spectra of terchebin indicated signals that were similar to those of the DHHDP group of geraniin. The second observation was that the H-1 of the phenazine **64**, derived from terchebin via **63**, shifted to a higher field. The shift was similar to that observed in geraniin.

#### 3.2.3. Isoterchebin

**Figure 27:**
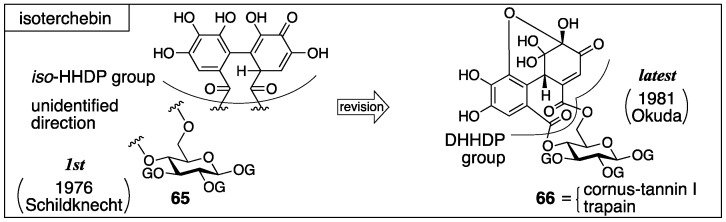
The transition of the structure of isoterchebin.

Isoterchebin was isolated from *Cytinus hypocisris* by Schildknecht and firstly given the structure **65** [59] (Figure 27). After that, Okuda isolated cornus-tannin I from *Cornus officinalis*, revealed its structure, and found that the structure of cornus-tannin I (**66**) was identical to isoterchebin. Consequently, at this time, the structure of isoterchebin was revised to be **66** [60]. Later, Nishioka isolated a tannin, trapain, from *Trapa japonica* FLEROV, which also had the structure **66** [61].

**Figure 28:**
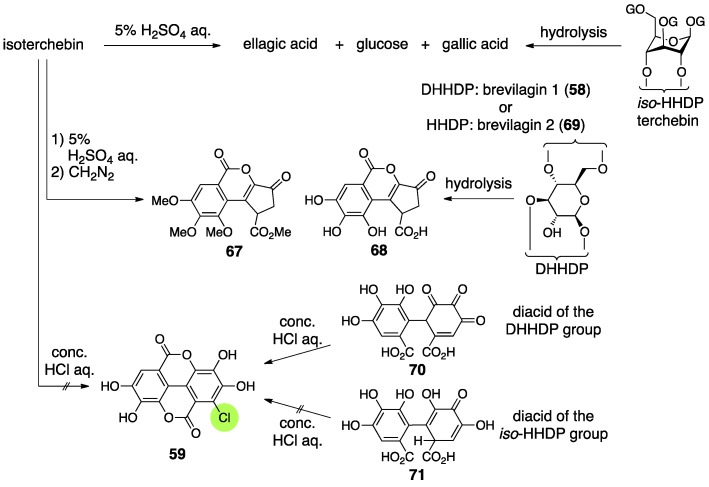
The transformations used for the determination of the structure **65**.

The structure **65** was determined by the combination of classic methods and NMR studies [59]. Hydrolysis with 5% sulfuric acid provided ellagic acid, d-glucose, and gallic acid (Figure 28). Meanwhile, one of the products derived by the methylation of the AcOEt extracts of the hydrolysates was **67** [62]. In those days, no ellagitannin containing structure **68** had been found. On the other hand, it was known that the hydrolysis of terchebin and brevilagins (**58** and **69**) [28], which had been considered to have the *iso*-HHDP and DHHDP groups, respectively, produced ellagic acid and **68** [63]. In addition, a method for the discrimination of the DHHDP and *iso*-HHDP groups had been known [52], which was that the treatment of dehydrohexahydroxydiphenic acid (**70**) with concentrated hydrochloric acid precipitated **59** out, but the treatment of **71** did not result in the same outcome. Because the hydrolysis of isoterchebin using concentrated hydrochloric acid did not provide **59**, Schildknecht considered that isoterchebin likely had the *iso*-HHDP group. From the ^1^H-NMR spectrum of isoterchebin, they identified the existence of three unsubstituted galloyl groups, a gallic acid moiety modified at the 2-position, a vinyl proton, phenolic hydroxy groups, and enolic hydroxy groups. Among them, the chemical shifts of the vinyl proton and enolic hydroxy groups were in good agreement with those of terchebin. Therefore, they assessed that isoterchebin had the *iso*-HHDP group on the basis of the structure **55** (Figure 23). The signals due to the glucose moiety also displayed that the pyranose was in the ^4^*C*_1_ form and the existence of a bridging group between O-4 and O-6. The observations collectively lead to the structure **65** [59].

**Figure 29:**
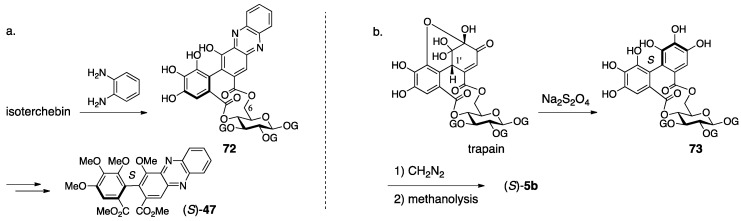
(**a**) The determination of the stereochemistry of the DHHDP group of isoterchebin (**66**) and (**b**) the reduction of trapain, which is the same compound with isoterchebin.

When Okuda isolated and determined the structure of cornus-tannin I, the structure that had been understood as the *iso*-HHDP group was revised to be the DHHDP group [53]. Accordingly, they doubted **65** that possessed the *iso*-HHDP group and concluded that cornus-tannin I and isoterchebin were the same compound because of their identical ^1^H-NMR spectra and physical properties. The NMR experiments revealed, in addition to Schildknecht’s observations, that three galloyl groups and one DHHDP group composed the molecule, that the DHHDP group was in the hemiacetal structure, and that no equilibrium mixture due to the existence of water occurred, which had been observed in geraniin. The condensation of isoterchebin with benzene-1,2-diamine provided the phenazine **72** (Figure 29a), which was transformed to known (*S*)-**47** [53] to exhibit the *S*-axial chirality. Hence, the chirality of the methine carbon of the DHHDP group was *S*. The decision that the phenazine formed on the O-6 side in structure **72** was attributed to the high-field shift of the C-6 of **72** compared to those of isoterchebin (−1.3 ppm) and **73** (−2.5 ppm) in the ^13^C-NMR. In addition, the conformational change due to the formation of the phenazine was exposed by the high- and low-field shifts of H-4 and H-6, respectively, in the transformation of isoterchebin to **72**.

Nishioka’s structural determination of trapain [61], which is the same compound with isoterchebin, was similar to that examined by Okuda [60]. The differences were in the method for disclosing the absolute structure of the DHHDP group and in the confirmation that the DHHDP group was in the six-membered hemiacetalic ring. Thus, the reduction of the DHHDP group of trapain using aqueous Na_2_S_2_O_4_ produced **73** (Figure 29b) [64]. Full methylation of **73** followed by methanolysis provided (*S*)-**5b**, the axial chirality of which was confirmed by optical rotation [42]. Therefore, the chirality of C-1′ in the DHHDP group was also *S*. For the confirmation of the six-membered hemiacetalic ring of the DHHDP group, long range proton decoupling was applied. Their discovery that the DHHDP group of trapain did not generate a mixture of isomers by the addition of water corresponded to previous discussions.

### 3.3. Correction Based on the Structure of the Chebuloyl Group: Chebulinic Acid and Chebulagic Acid

**Figure 30:**
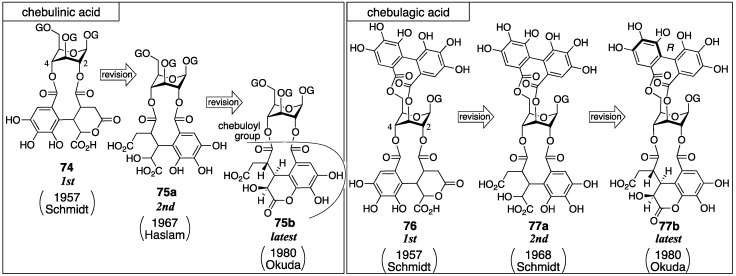
The transition of the structures of chebulinic acid and chebulagic acid.

Chebulinic acid and chebulagic acid are the major ellagitannins of myrobalans (fruit of *Terminalia chebula*) [65]. Fridolin isolated chebulinic acid for the first time in 1884 [18]. From this plant, terchebin was also isolated (Section 3.2.2). The first structures of chebulinic acid (**74**) and chebulagic acid (**76**) were determined by Schmidt (Figure 30) [57,58]. Subsequently, Haslam revised **74** to be **75a** [66]. Accordingly, Schmidt revised **76** to be **77a** [28]. These structures were revised once more by Okuda to be **75b** and **77b** [67].

**Figure 31:**
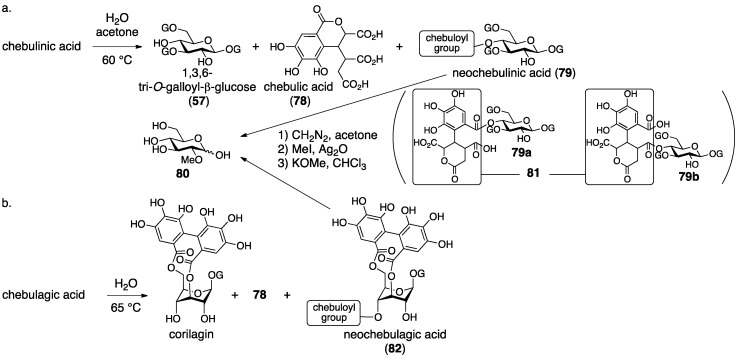
(**a**) The hydrolysis of chebulinic acid and the determination of the position of the chebuloyl group and (**b**) the hydrolysis of chebulagic acid.

Schmidt’s structural determination of **74** and **76** is based on decomposition experiments. The hydrolysis of chebulinic acid provided the known 1,3,6-tri-*O*-galloyl-β-glucose (**57**) [54] and chebulic acid (**78**) [68] (Figure 31a) along with a new compound named neochebulinic acid (**79**, C_41_H_34_O_28_ by elemental analysis). The successive full methylation of **79** and hydrolysis produced 2-*O*-methylglucose (**80**). Therefore, **79** is a seco acid with an unmodified 2-OH of glucose. With the authors’ consideration that the chebuloyl group was an oxidized HHDP group [65] and with the structure of **78** in mind, the authors gave the structure **81** for the chebuloyl group. The determination suggests structures **79a** and **79b** for neochebulinic acid. They adopted **79a** considering that the aliphatic ester, which is more reactive, was hydrolyzed swiftly [58]. As the structure of chebulinic acid is lactonized **79a** at O-2, it was **74**. On the other hand, the hydrolysis of chebulagic acid released corilagin [69], **78**, and a halfway hydrolysate named neochebulagic acid (**82**) that possessed unmodified 2-OH (Figure 31b). These products indicated structure **76** [57].

**Figure 32:**
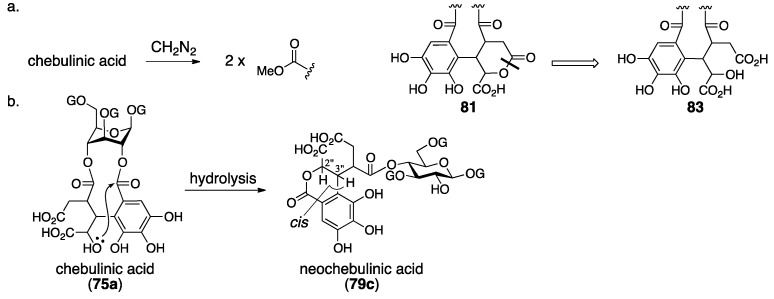
The consideration of the structural revision to the second structures of chebulinic acid and chebulagic acid.

Haslam observed two signals indicating methoxycarbonyl groups on a ^1^H-NMR spectrum of diazomethane-treated chebulinic acid (Figure 32a). The result could not be explained with structure **74** that bore only one carboxy group. Hence, the structure of the chebuloyl group was not **81**, but was supposed to be non-lactonized **83**. In addition, they considered that the intramolecular lactonization due to the nucleophilic attack of a hydroxy group in the chebuloyl group produced neochebulinic acid (Figure 32b). With the fact that the ester bond on the O-2 of glucose was easier to cleave, they proposed structure **75a** [66]. After that, Schmidt revised the structure of **76** to **77a** according to Haslam’s revision [28]. In the same report, Schmidt mentioned the *cis*-relationship between H-2″ and H-3″ of **79c** on the basis of the ^1^H-NMR coupling constant.

**Figure 33:**
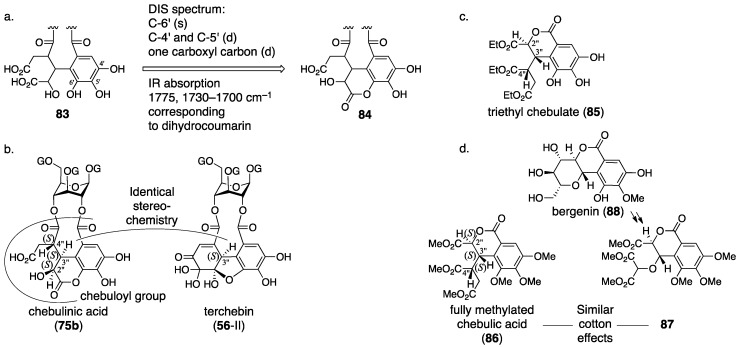
The structural revision of the chebuloyl group for the latest structures of chebulinic acid (**75b**) and chebulagic acid (**77b**). DIS, differential isotope shift.

Okuda re-examined the structure of chebulinic acid using the DIS method [51] that had been effective in the structural determination of geraniin (Section 3.2.1) [23]. Consequently, they revealed that only C-4′ and C-5′ had the structure of “C–OH” among the aromatic carbons bearing an oxygen atom in the chebuloyl group (Figure 33a). Since the revelation could not be explained with structure **83**, they conceived structure **84** that was a lactone mediated by O-6′. Structure **84** was ensured by three facts: (1) the DIS spectrum disclosed that only one of the four –CO_2_– structures in the chebuloyl group was –CO_2_H; (2) only one signal shifted to the lower magnetic field in the ^13^C-NMR spectrum of the Et_3_N salt of chebulinic acid; and (3) the reported IR absorptions (1775 and 1730–1700 cm^–1^) by Haslam were relevant to the dihydrocoumarin ring [66]. Thus, the structure of chebulinic acid was elucidated to be **75b**, and, similarly, the structure of chebulagic acid was revised to be **77b**. However, these structures might not generate “the two methoxy carbonyl groups”, which Haslam previously observed on the methylated chebulinic acid. Okuda considered the inconsistency and concluded that the lactone of **84** was easy to cleave by treatment with diazomethane, which allowed for the formation of the two methyl esters. The stereochemistry indicated in structures **75b** and **77b** is based on the following consideration. Because chebulinic acid and chebulagic acid were isolated from the same plant from which terchebin (Section 3.2.2) was isolated, the stereochemistry of the C-3″ position was estimated to be *S*, that is, the same stereochemistry of C-3″ in terchebin (Figure 33b). The relative configuration of chebulic acid (**78**) was determined by an X-ray diffraction study of triethyl chebulate (**85**) (Figure 33c) [70]. The absolute configuration of **78** was confirmed to be *2″S*, *3″S*, *4″S* on the basis of the comparison of the CD spectrum of fully methylated chebulic acid (**86**) [71] to that of **87**, derived from bergenin (**88**), of which the absolute structure had been known (Figure 33d) [72,73]. The result indicated that the absolute stereochemistries of chebulinic acid and chebulagic acid were *2″S*, *3″S*, *4″S*. The axial chirality of the HHDP group of chebulagic acid was identified to be *R* because chebulagic acid contained corilagin, of which the axial chirality of the HHDP group was known to be *R* [23].

### 3.4. Compounds Containing a C–C-Connected Trimer and Tetramer of the Galloyl Group

#### 3.4.1. Castalin, Vescalin, Castalagin, Vescalagin, Casuarinin, and Stachyurin

**Figure 34:**
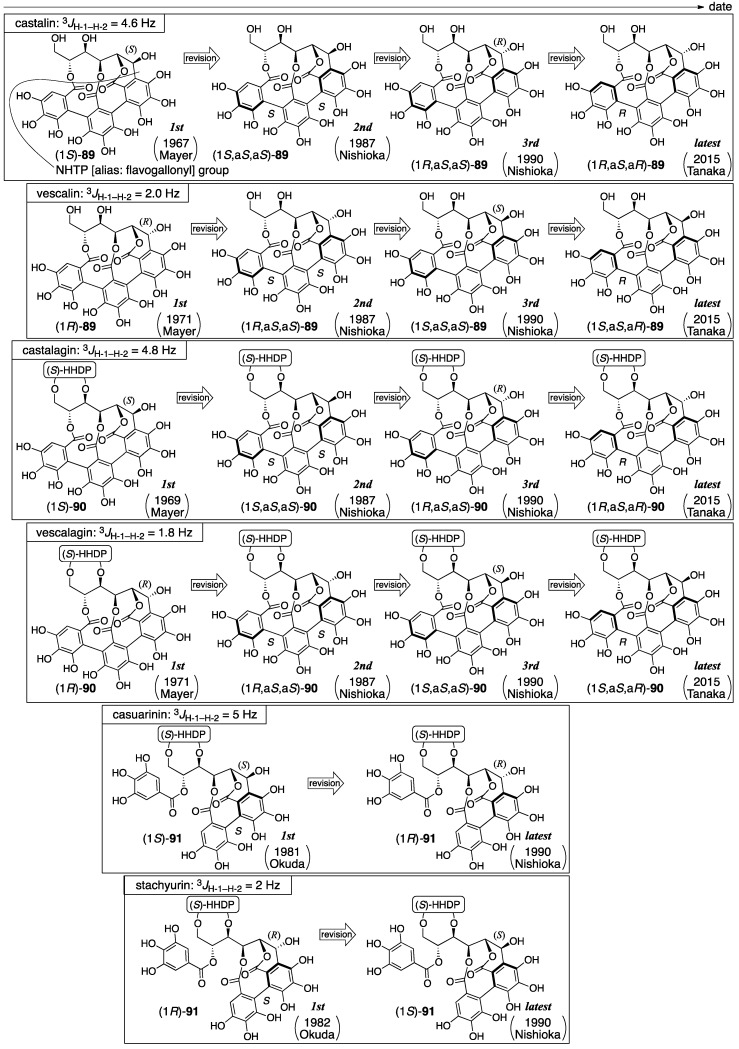
The transition of the structure of castalin, vescalin, castalagin, vescalagin, casuarinin, and stachyurin.

All of the six ellagitannins listed above have a *C*-glycosidic bond in common (Figure 34). Mayer isolated castalin and vescalin from *Castanea sativa* and *Quercus sessiliflora* in 1967 [74] and gave their first structures as (1*S*)-**89** and (1*R*)-**89**, respectively, which comprised the NHTP group [75,76]. They also isolated castalagin and vescalagin from the same plants [74] and gave their first structures as (1*S*)-**90** and (1*R*)-**90**, respectively [77,78]. Subsequently, Okuda isolated casuarinin and stachyurin from *Casuarina stricta* and *Stachyurus praecox* and reported the respective structures (1*S*)-**91** and (1*R*)-**91** [37,79]. In 1987, Nishioka determined the axial chirality of the NHTP group to be *S,S* and reported the second structures for castalagin and vescalagin [80]. Thus, the axial chirality was also added to the structures of castalin and vescalin, resulting in their second structures. After that, Nishioka corrected the stereochemistries of the 1-OH of casuarinin and stachyurin, which lead their latest structures, (1*R*)-**91** and (1*S*)-**91**, respectively [81]. In accordance with the correction, the C-1 stereochemistries of castalin, vescalin, castalagin, and vescalagin were revised, resulting in their third structures. Twenty-five years later, in 2015, Tanaka corrected the axial chirality of the NHTP group and revised the structures of castalin and vescalin to be (1*R*,a*S*,a*R*)-**89** and (1*S*,a*S*,a*R*)-**89**, respectively [82]. Consequently, the structures of castalagin and vescalagin were revised, resulting in the latest structures. In 2017, Quideau synthesized the latest structure of vescalin [83].

The first structure of castalin was revealed on the basis of the decomposition of the natural product and NMR studies [75]. The decomposition of castalin with hydrochloric acid and MeOH followed by the treatment of the decomposed products with diazomethane gave structurally unknown derivatives. A fragment ion peak in the mass spectrum of the derived compound indicated the existence of the NHTP group. Chemical shifts of the ^1^H-NMR spectrum of castalin showed that (1) the NHTP group combined with a glucose moiety at O-2, O-3, and O-5 through ester bonds and (2) there was a *C*-glycosidic bond at C-1. The *S*-stereochemistry of C-1 was ascribed to the coupling constant of 4.6 Hz for ^3^*J*_H-1–H-2_.

**Figure 35:**
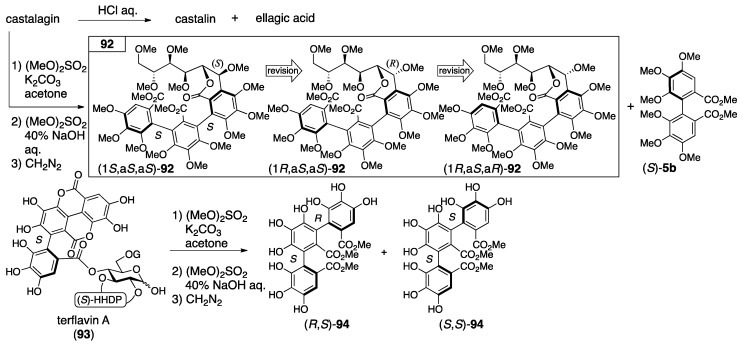
The hydrolysis of castalagin, and the determination of the axial chiralities in the second structure of castalagin.

The first structure of castalagin was determined on the basis of the first structure of castalin, (1*S*)-**89** [77]. The hydrolysis of castalagin yielded castalin [74,75] and ellagic acid (Figure 35). Hence, castalagin has the structure (1*S*)-**90** that is composed of the HHDP group and castalin.

The first structures of vescalin and vescalagin were disclosed in a manner similar to that above [76,78]. Because the hydrolysis of vescalagin provided vescalin and ellagic acid, structure (1*R*)-**90** was illustrated. The C-1 *R*-stereochemistry was given according to the coupling constant of 1.8 Hz for ^3^*J*_H-1–H-2_.

In the determination of the axial chirality of the NHTP group that lead to the second structure of castalagin, CD spectra were used [80]. Thus, starting from castalagin, the three-step transformation introduced **92** along with (*S*)-**5b** (Figure 35). On the other hand, terflavin A (**93**), of which the absolute stereochemistry had been known [84], was converted to (*R*,*S*)-**94** and (*S*,*S*)-**94** in three steps. Of these, the optically inactive (*R*,*S*)-**94** was the *meso*-isomer. The axial chirality of the optically active isomer was (*S*,*S*)-**94** since the relevant part in **93** was *S*. The CD spectra of **92** showed a similar waveform to that of (*S*,*S*)-**94** in which the Cotton effect was negative at around 230 nm and positive at around 252 nm. According to the observation, the axial chirality of the NHTP group in **92** was determined as being *S*,*S*; hence, the structure was (1*S*,a*S*,a*S*)-**92**. The heating of an aqueous solution of vescalagin gave rise to the isomerization at C-1 to produce castalagin [74]; therefore, the stereochemistry of the NHTP group of vescalagin was the same as *S*,*S* and, thus, the structure was (1*R*,a*S*,a*S*)-**90** [80]. Similarly, as castalin and vescalin were structural parts of castalagin and vescalagin [80], these second structures might be revised to (1*S*,a*S*,a*S*)-**89** and (1*R*,a*S*,a*S*)-**89** at this time despite its absence in the literature.

**Figure 36:**
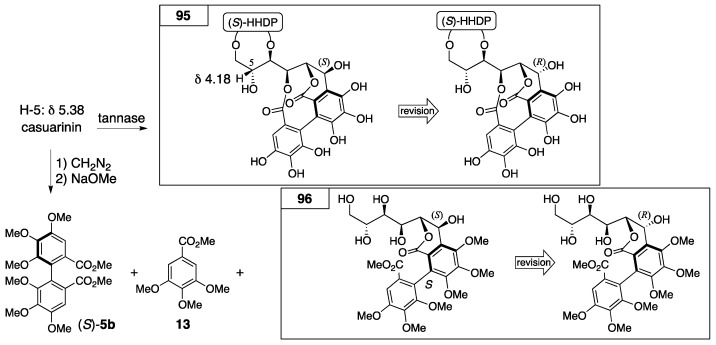
The transformations used for the determination of the first structure of casuarinin ((1*S*)-**91**).

The first structures of casuarinin and stachyurin were introduced by decomposition and NMR studies [37]. The tannase hydrolysis of casuarinin provided **95** (Figure 36). By the transformation, the ^1^H-NMR chemical shift of H-5 shifted to a higher magnetic field. In addition, because the coupling constant was 5 Hz for the ^3^*J*_H-1–H-2_ of casuarinin (Figure 34), the stereochemistry of C-1 was demonstrated to be *S* according to the structure of castalin ((1*S*)-**89**). On the other hand, the sequential treatment of casuarinin with diazomethane followed by the methanolysis of the product gave (*S*)-**5b**, **13**, and **96**. The CD spectrum of **96** partially indicated a similar Cotton effect to that of (*S*)-**5b**. Therefore, the *S*-axial chirality in **96** was provided. In the determination of the first structure of stachyurin ((1*R*)-**91**), the following two facts were the conclusive factors: (1) the ^3^*J*_H-1–H-2_ value was 2 Hz; and (2) the heating of the aqueous solution of casuarinin caused the occurrence of isomerization to yield stachyurin [79].

**Figure 37:**
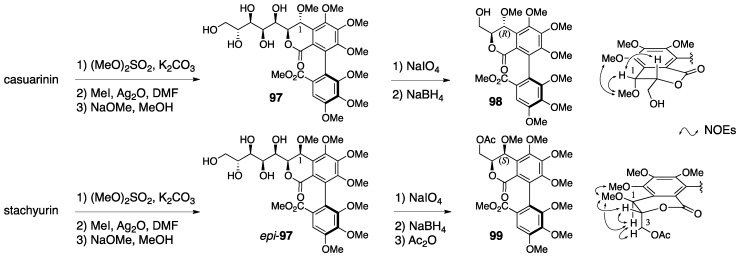
The grounds for swapping the stereochemistry of C-1 to lead to the latest structures of casuarinin and stachyurin. NOE, nuclear Overhauser effect.

In the structural revision to the latest structures of casuarinin and stachyurin, nuclear Overhauser effect (NOE) difference spectroscopy played a key role. The natural products casuarinin and stachyurin were introduced to **98** and the acetate **99** through **97** and *epi*-**97**, respectively (Figure 37) [81]. The NOE difference spectrum of **99** indicated a *cis*-configuration at H-1 and C-3. Therefore, the stereochemistry of C-1 was *S*. By contrast, there was no decisive NOE relationship in **98** for the determination of the stereochemistry of C-1, but it was the epimer at C-1; hence, the stereochemistry of **98** was *R*.

The correction of the C-1 stereochemistry of casuarinin and stachyurin affected the structures of castalagin and vescalagin. Thus, on the basis of the similarity of the coupling constants (^3^*J*_H-1–H-2_) (Figure 34), the C-1 stereochemistries of castalagin and vescalagin were changed to be *R* and *S*, respectively; hence, the structures were revised to be (1*R*,a*S*,a*S*)-**90** and (1*S*,a*S*,a*S*)-**90** (the third structures). Likewise, despite their absence in the literature, the structures of castalin and vescalin could be revised to be their third structures (1*R*,a*S*,a*S*)-**89** and (1*S*,a*S*,a*S*)-**89**, respectively, where the stereochemistry at C-1 was swapped.

**Figure 38:**
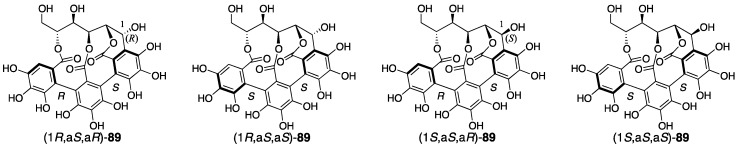
The computer-calculated compounds that led to the structural revisions of castalin and vescalin to the latest structures.

In the correction of the axial chirality of the NHTP group in 2015, chemical calculations were utilized [82]. Using the density functional theory (DFT) method, Tanaka calculated each electronic circular dichroism (ECD) spectrum of four isomers of the castalin/vescalin **89** (Figure 38), in which the C-1 stereochemistry and the axial chirality of the NHTP group differed. As a result, the calculated spectrum for (1*R*,a*S*,a*R*)-**89** and the actual spectrum of castalin [75] were in good agreement. Similarly, the calculated data for (1*S*,a*S*,a*R*)-**89** were similar to the measured spectra of vescalin [76]. As castalin and vescalin were the hydrolysates of castalagin and vescalagin, respectively [74], these structures were also revised to be (1*R*,a*S*,a*R*)-**90** and (1*S*,a*S*,a*R*)-**90** [82]. In the report, Tanaka points out that the correction of the axial chirality would affect the structures of the other natural ellagitannins containing the NHTP group, such as roburins A−D [85], castaneanins A−D [86], acutissimins A (Figure 1) and B [14], anogeissusins A and B [87], anogeissinin [87], and several related metabolites, such as mongolicains A and B [88].

**Figure 39:**
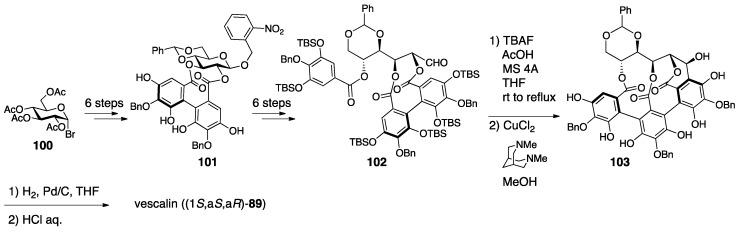
The first total synthesis of vescalin.

Quideau’s synthesis of vescalin was achieved by the development of a novel method for synthesizing the NHTP group (Figure 39) [83]. The six-step transformation of **100** provided **101** that possessed the (*S*)-HHDP group between O-2 and O-3. An additional six-step transformation, including hydrolysis, oxidation at the anomeric position, and the introduction of the galloyl group to O-5, gave **102**. The removal of the TBS groups from **102** triggered the *C*-glycosylation. Oxidation by CuCl_2_ and *N*,*N*′-dimethylbispidine constructed the NHTP group to provide **103**. Further hydrogenolysis of the benzyl groups and hydrolysis of the benzylidene acetal afforded vescalin. The synthetic method for the NHTP group developed in the total synthesis showed, for the first time, that the group could be chemically synthesized using the oxidative coupling of phenols.

#### 3.4.2. Punicalin and Punicalagin

**Figure 40:**
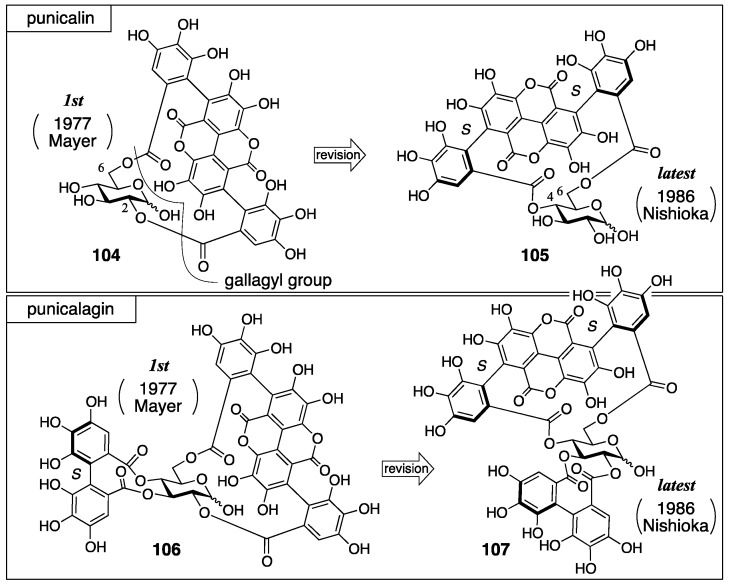
The transition of the structures of punicalin and punicalagin.

Punicalin and punicalagin were isolated from pomegranate (*Punica granatum*) their first structures, **104** and **106**, were reported by Mayer in 1977, in which the gallagyl group bridged between the O-2 and O-6 of glucose (Figure 40) [89]. Nishioka revised the structures to **105** and **107** in 1986 [90]. In the revision, the bridging position of the gallagyl group was altered and the axial chirality was determined.

**Figure 41:**
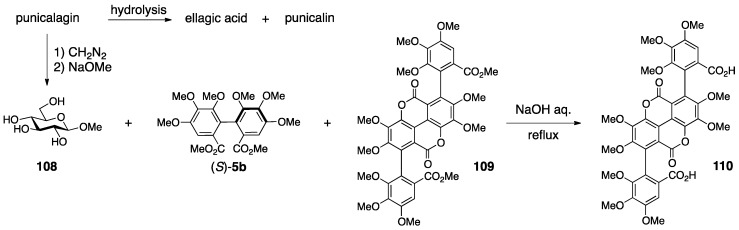
The degradation used for the structural determination of the first structures of punicalin and punicalagin.

The first structures were determined using degradation (Figure 41), NMR studies, and the consideration of molecular models. The hydrolysis of punicalagin provided ellagic acid and punicalin. Therefore, punicalin was a component of punicalagin. The treatment of punicalagin with diazomethane and the subsequent methanolysis produced **108**, (*S*)-**5b** [77], and **109**. The hydrolysis of **109** yielded dicarboxylic acid **110**. On the basis of the ^1^H-NMR and mass spectra, structures **109** and **110** were determined. Taken together, Mayer supposed that punicalagin had a hydroxy group on the C-1 of glucose and that there were HHDP and gallagyl groups on the other oxygens. The fact that punicalin and punicalagin were negative to the qualitative analysis using aniline hydrogen phthalate [19] and the knowledge that 2-*O*-galloyl glucose is basically negative to detection [91] lead to the prediction that the O-2 of both punicalin and punicalagin was esterified. The other connecting site, O-6, was revealed according to the consideration that the gallagyl group could connect only with O-6 in assembling a molecular model. Thus, the HHDP group bridged between the residual O-3 and O-4 in the structure of punicalagin. Later, Schilling reported that the glucose moieties in punicalin and punicalagin were both in the ^4^*C*_1_ form on the basis of the ^1^H-NMR coupling constants [92].

**Figure 42:**
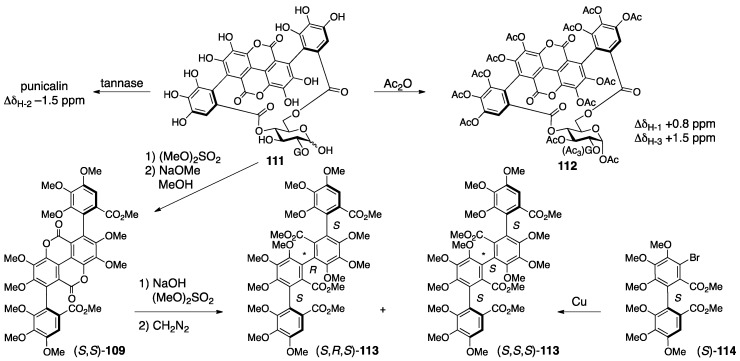
The transformations that led to the latest structures of punicalin and punicalagin.

The revision to the latest structures was triggered by the discovery of the related natural tannin **111** (no specific name) (Figure 42) [90]. First, the connecting positions of the gallagyl group in punicalin and punicalagin were revised. The ^1^H-NMR spectrum of **111** indicated the existence of one galloyl group. The tannase-mediated hydrolysis of **111** produced punicalin where the ^1^H-NMR chemical shifts of the H-2 shifted to a higher field (Δδ −1.5 ppm). After the acetylation of **111**, the ^1^H-NMR chemical shifts of the H-1 and H-3 of the obtained **112** were in a lower field than those of **111**, which demonstrated that O-1 and O-3 were acetylated. The above led to the conclusion that **111** possessed hydroxy groups on C-1 and C-3, a galloyl group on O-2, and a gallagyl group bridging between O-4 and O-6. The fact that punicalin was a hydrolysate of **111** meant that the gallagyl group bridged between O-4 and O-6. Since the position of the gallagyl group in punicalin was the same in punicalagin, the position of the HHDP group in punicalagin was revised to be between O-2 and O-3. Then, the *S*,*S*-axial chiralities in the gallagyl group were given. Thus, the methylation of **111** and the subsequent methanolysis released (*S*,*S*)-**109**, which was converted to (*S*,*R*,*S*)-**113** and (*S*,*S*,*S*)-**113** by additional two steps, although the axial chiralities were unknown at this point. On the other hand, the Ullmann coupling of the known (*S*)-**114** [43] yielded (*S*,*R*,*S*)-**113** and (*S*,*S*,*S*)-**113**, which were identical to those derived from **109**. Hence, these axial chiralities were *S*,*R*,*S* and *S*,*S*,*S* from which the disappearance of the middle axial chirality due to bislactonization revealed the *S*,*S*-structure.

### 3.5. Correction Based on the Bonding Position of C–O-Connected Components

#### 3.5.1. Sanguiin H-2, H-3, and H-6

**Figure 43:**
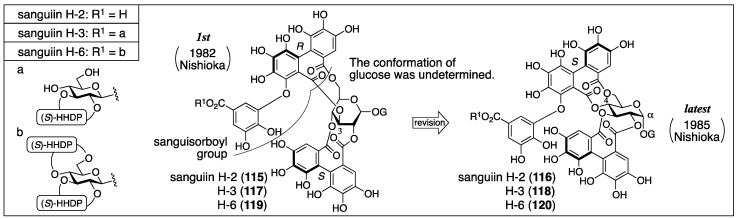
The transition of the structure of sanguiin H-2, H-3, and H-6. The conformation of the glucose moiety in the first structures was undetermined.

Sanguiin H-2, H-3, and H-6 were isolated from great burnet (*Sanguisorba officinalis*) and given their respective first structures by Nishioka (Figure 43) [93,94]. Three years later, Nishioka revised them to the latest structures, in which the bridging positions were altered [43], the axial chirality of the sanguisorboyl group was changed, and the α-stereochemistry was determined for the anomeric gallate.

**Figure 44:**
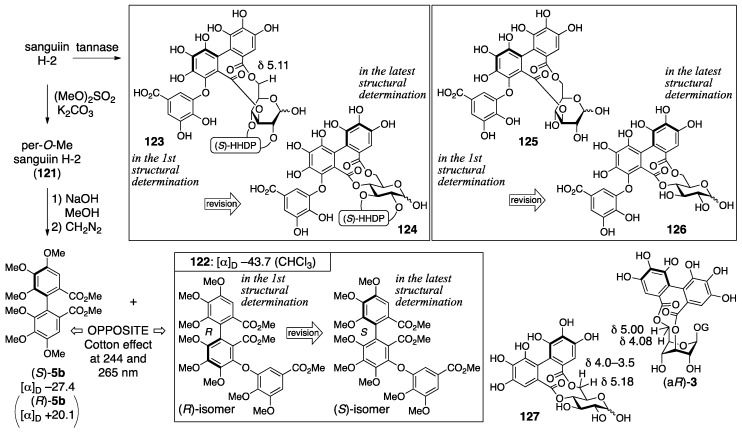
The observations used for the structural determination of sanguiin H-2 (**115**). The conformation of the glucose moiety of **123** and **125** was undetermined.

The process for the determination of the first structure of sanguiin H-2 (**115**) consisted of transformations of the natural product and NMR studies (Figure 44) [93]. The methylation of sanguiin H-2 provided the per-*O*-methylated **121**, the mass spectrum of which indicated the existence of sanguisorboyl, HHDP, and galloyl groups. The ^1^H-NMR of **121** exhibited signals for seven hydrogens on aromatic rings; thus, each one of the sanguisorboyl, HHDP, and galloyl groups was contained in sanguiin H-2. The methanolysis of **121** and the subsequent treatment with diazomethane produced (*S*)-**5b** and **122**. The *S*-axial chirality was provided by a comparison of the specific optical rotation to that of the known (*R*)-**5b** [42]. The reason for the *R*-axial chirality of **122** was the opposite Cotton effect observed at 244 and 265 nm in the CD spectra of (*S*)-**5b** and **122**. The hydrolysis of sanguiin H-2 with tannase provided two products. One was an anomeric mixture bearing the sanguisorboyl and HHDP groups; hence, the galloyl groups were situated on O-1 in sanguiin H-2. The other was determined to be **125**, which possess a 3,6-*O*-bridged structure. The bridge position was revealed by a comparison of its ^1^H-NMR spectra to those of the known (a*R*)-**3** [31] and **127** [64]. Embodying the information presented structure **115**.

**Figure 45:**
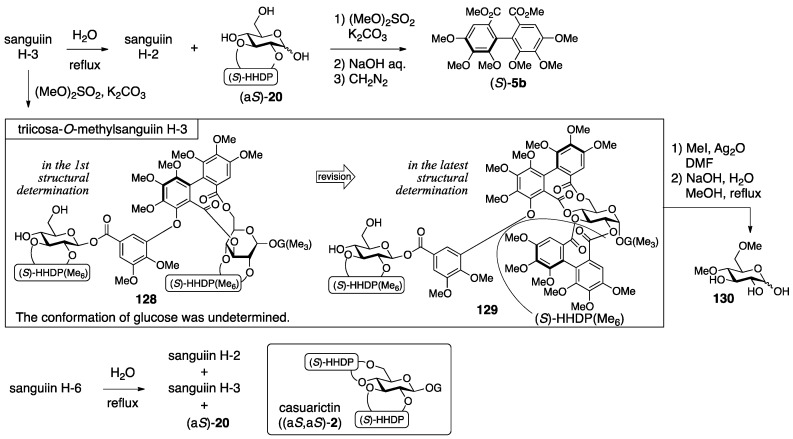
The transformations that led to the first structures of sanguiin H-3 (**117**) and H-6 (**119**). The conformation of the glucose moiety of **128** was undetermined.

The structural determinations for sanguiin H-3 (**117**) and H-6 (**119**) were proceeded similarly [93,94]. The ^1^H/^13^C-NMR spectrum of sanguiin H-3 indicated that a glucose derivative possessing one HHDP group had combined with sanguiin H-2. The methylation of sanguiin H-3 provided triicosa-*O*-methylsanguiin H-3 (Figure 45); further methylation and subsequent hydrolysis released 4,6-di-*O*-methyl-d-glucose (**130**). In addition, the partial hydrolysis of sanguiin H-3 provided sanguiin H-2 and (a*S*)-**20**. The *S*-axial chirality of the HHDP group in (a*S*)-**20** was confirmed by the transformation of it to (*S*)-**5b**. By integrating these pieces of information, structure **117** was determined. The ^1^H-NMR spectrum of sanguiin H-6 demonstrated a structure that had one more HHDP group merged with sanguiin H-3. The partial hydrolysis of sanguiin H-6 gave sanguiin H-2, sanguiin H-3, and (a*S*)-**20**. The determination of the *S*-axial chirality of the 4,6-*O*-HHDP in **119** relied on the similarity of the ^13^C-NMR spectrum of sanguiin H-6 and casuarictin ((a*S*,a*S*)-**2**).

**Figure 46:**
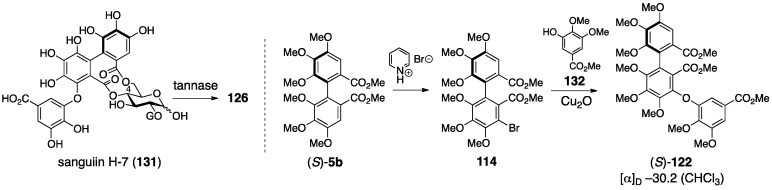
The information referred to in the structural revision to **116**.

The structural revision to the latest structures is founded on identification and synthesis [43]. First, the hydrolysate of sanguiin H-7 (**131**) (Figure 46), which possessed the sanguisorboyl group between O-4 and O-6, was identical to the tannase hydrolysate derived from sanguiin H-2 (see also Figure 44). Therefore, the bridging position of the sanguisorboyl group in the hydrolysate was corrected to be between O-4 and O-6 as in **126**. Due to this correction, the position of the HHDP group in sanguiin H-2 was altered to be between O-2 and O-3 from between O-2 and O-4. Then, the axial chirality of the sanguisorboyl group was revised from *R* to *S*. Thus, the Cu_2_O-mediated coupling [95] of **114**, which was a brominated (*S*)-HHDP compound derived from (*S*)-**5b**, and the gallic acid derivative **132** provided (*S*)-**122**. The ^1^H-NMR spectrum and the sign of the specific optical rotation of (*S*)-**122** were identical to those of **122** derived from **121** (Figure 44). The stereochemistry of the anomeric position was determined to be α on the basis of the ^1^H-NMR coupling constant (^3^*J*_H-1–H-2_ = 4 Hz). Hence, the structure of sanguiin H-2 was **116**. The structures of sanguiin H-3 and H-6 were similarly revised to be **118** and **120**, respectively.

#### 3.5.2. Alnusiin

**Figure 47:**
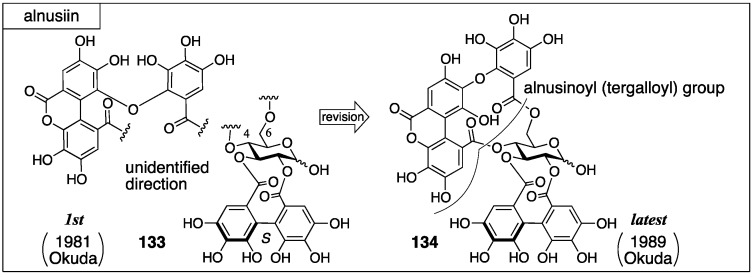
The transition of the structure of alnusiin.

Alnusiin was isolated from the fruits of *Alnus sieboldiana* by Okuda in 1981. Initially, the structure **133** was advocated (Figure 47) [96]. In the structure, the direction of the bridging component between O-4 and O-6 of the glucose moiety was unspecified. The structure **133** was revised to **134** in 1989 by Okuda [97]. The structure of the partly lactonized macaranoyl group in **133** had included an error. The component was a partly lactonized tergalloyl group.

**Figure 48:**
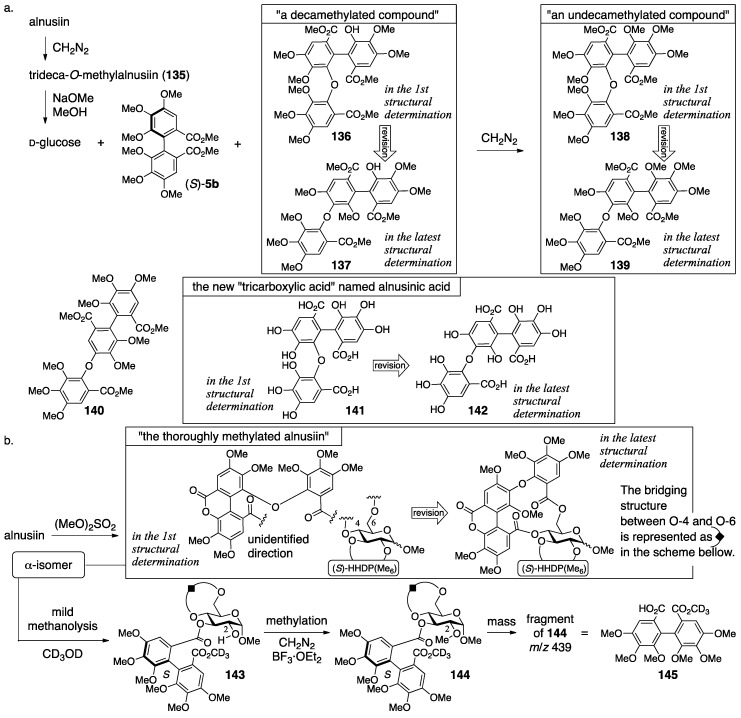
The information that led to **133**.

In the process leading to **133**, several types of methylation were effectively used [96]. The observation of two anomeric carbons in ^13^C-NMR revealed that alnusiin was a mixture of anomers. The methylation of the phenolic hydroxy groups of alnusiin produced trideca-*O*-methylalnusiin (**135**) (Figure 48a). The methanolysis of **135** provided d-glucose, (*S*)-**5b**, and a decamethylated compound. The further methylation of this provided an undecamethylated compound whose mass spectrum was similar to that of known **140**, but these ^1^H-NMR spectra were different [98]; hence, the “tricarboxylic acid” was a new compound and named alnusinic acid. Structure **141**, composed of the macaranoyl-group skeleton, was determined according to the additivity of the substituent effect in the ^13^C-NMR spectrum. The facts that the product obtained by methanolysis of **135** was not an “undecamethylated compound” but a “decamethylated compound” and that one of the carbonyl carbons appeared with an upfield shift (δ 164.1) in the ^13^C-NMR indicated the existence of the lactone. Therefore, the fragment that consisted of three condensed galloyl groups was called the alnusinic acid monolactone group. The conformation of the glucose moiety in the thoroughly methylated alnusiin (Figure 48b), which was prepared by the treatment of alnusiin with dimethyl sulfate, was ^4^*C*_1_ according to the ^1^H-NMR coupling constants. The conformation limited the positions of the bridges to be between O-2 and O-3 and between O-4 and O-6. The mild methanolysis of the α-isomer of the thoroughly methylated alnusiin with CD_3_OD cleaved the ester bond on O-2 of the glucose, which was confirmed by the disappearance of the esterification shift, to give **143**. The subsequent methylation produced the O-2-metylated compound **144**. The molecular weight of a fragment observed in the mass spectrum of **144** was 439, which corresponded to that of the HHDP derivative **145**. Accordingly, the HHDP group bridged between O-2 and O-3. The other component, which was the alnusinic acid monolactone group, bridged between O-4 and O-6.

**Figure 49:**
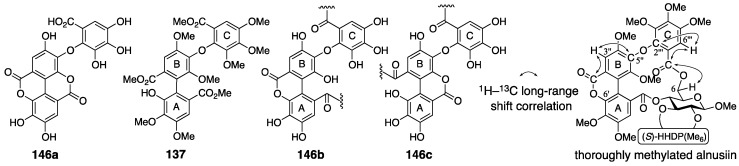
The compounds used for considering the latest structure of alnusiin **134**.

In the revision to the latest structure, the component bridging between O-4 and O-6 was changed from a derivative of the macaranoyl group to that of the tergalloyl group [97,99]. The hydrolysate of alnusiin was found to be **146a** (Figure 49). Methanolysis of the trideca-*O*-methylalnusiin (**135**) yielded **137**. These results limit the structure of the fragment to only **146b** and **146c**. An NMR analysis observing the ^1^H–^13^C long-range shift correlations showed a cross-peak between H-3″ on the B-ring and a carbonyl carbon of lactone (see the thoroughly methylated alnusiin); hence, it was determined to be **146b**. The long-range correlation experiment also displayed cross-peaks between H-6′′′ and C-2′′′ and between H-6′′′ and a carbonyl carbon. In addition, the carbonyl carbon exhibited a correlation with the H-6 of glucose. These correlations are the conclusive evidence for the determination of the direction of the alnusinoyl (that is alnusinic acid monolactone) group in the structure of **134**. Note that the alnusinoyl group has the same framework as the tergalloyl group. The alnusinoyl group’s name likely refers to the lactonized structure, but the name is used only in this report to the best of our knowledge. There is a report that calls the lactone fragment “the monolactonized tergalloyl group” [99].

#### 3.5.3. Alnusnin A and B

**Figure 50:**
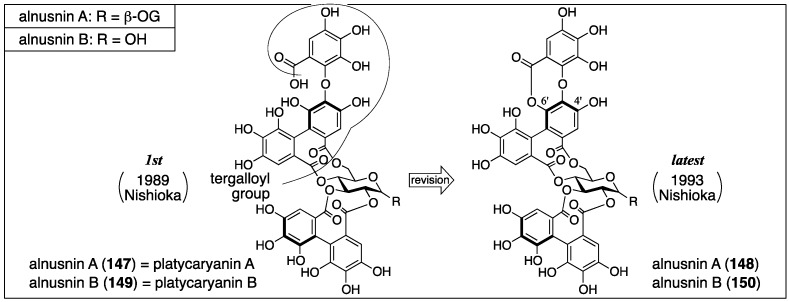
The transition of the structure of alnusnin A and B.

Alnusnin A and B were first isolated from *Alnus sieboldiana* and given their first structures **147** and **149**, respectively, by Nishioka in 1989 (Figure 50) [100]. In 1993, Nishioka isolated platycaryanin A and B. The determined structures of platycaryanins were **147** and **149**, respectively. Because platycaryanin A and B were not identical to alnusnin A and B, they reconsidered the structures. Each of the revised structures **148** and **150** contains a lactonized tergalloyl group [101].

**Figure 51:**
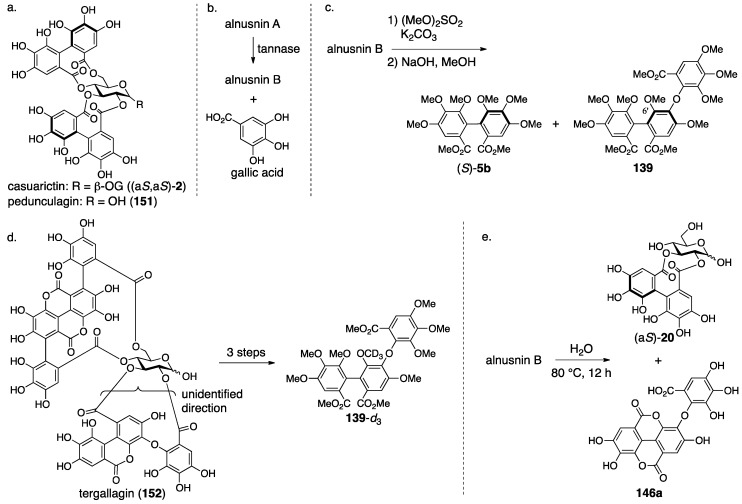
The transformations used in the determination of the first structures of alnusnin A (**147**) and B (**149**).

In the determination of the first structures of alnusnin A and B (**147** and **149**) [100], the presence of the galloyl, HHDP, and “triphenoyl” groups in alnusnin A were suggested by the ^1^H/^13^C-NMR spectra. Here, the “triphenoyl” means a component that has three hydroxylated benzoyl groups. The spectra of alnusnin A indicated that the chemical shifts and coupling patterns of sugar signals were in good agreement with those found in casuarictin ((a*S*,a*S*)-**2**) (Figure 51a) [47]. The hydrolysis of alnusnin A by tannase provided alnusnin B and gallic acid (Figure 51b). The FAB-MS spectra of alnusnin A and B supported their structures in terms of their molecular weights. The presence of the HHDP and triphenoyl groups in alnusnin B was indicated by the ^1^H/^13^C-NMR spectra with the information that the glucose moiety was in a hemiacetal form. The spectra also exhibited evidence that the signals, due to the sugar moiety, were similar to those found in the known **151**, of which the conformation of the glucose moiety was ^4^*C*_1_. The information demonstrated that the HHDP and the triphenoyl groups were attached to the 2,3- and 4,6-positions of the glucose moiety. The structures of the HHDP and the triphenoyl groups were determined by comparison of (*S*)-**5b** and **139** (Figure 51c), which is derived from alnusnin B, through the full-methylation of the hydroxy groups and subsequent methanolysis. The axial chirality of the (*S*)-HHDP group was provided by comparison of the optical rotation of **5b** derived from alnusnin B to the known **5b** [42]. The triphenoyl group was determined to be the tergalloyl group on the basis of the identical ^1^H-NMR signals of **139** derived from alnusnin B to those of **139**-*d*_3_ derived from a known compound, tergallagin (**152**), except for OCH_3_ on C-6′ (Figure 51d) [84]. The *S*-axial chirality of the tergalloyl group was estimated by the similarity of its CD spectrum to that of the known (*S*)-**5b**. The 2,3-location of the (*S*)-HHDP group in alnusnin B was established by the fact that the partial hydrolysis of alnusnin B yielded the known (a*S*)-**20** [31] together with **146a** (Figure 51e) [84].

The discovery of platycaryanins A and B in 1993 triggered the structural revision of alnusnins A and B [101]. The structures **147** and **149** were given for the structures of platycaryanins A and B, respectively, on the basis of NMR studies and transformation to known compounds. Despite the identical structures of platycaryanins and alnusnins, their NMR spectra were different. Thus, the authors reinvestigated the structures of alnusnin A and B. In the reinvestigations, the FAB-MS of alnusnin A and B showed their molecular weights, both of which were 18 smaller than the molecular weights of structures **147** and **149**. Therefore, the authors supposed the lactone forms **148** and **150**. Lactonization was possible at O-4′ and O-6′, from which the position was determined to be O-6′ according to the ^13^C-NMR chemical shifts and the ^1^H–^13^C long-range correlation spectroscopy (COSY) spectrum. The supposition was confirmed by the fact that the lactonization of platycaryanin B (**149**) using EDCI·HCl provided alnusnin B (**150**).

**Figure 52:**
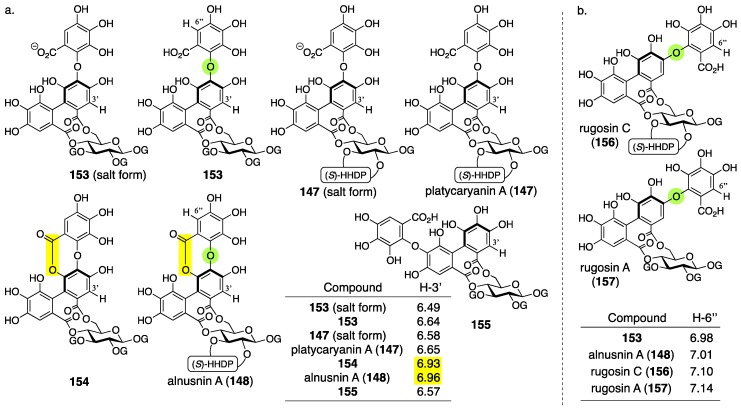
The notable difference of the ^1^H-NMR chemical shift observed (**a**) between the lactonized and non-lactonized forms of the tergalloyl moieties and (**b**) between the tergalloyl and valoneoyl moieties. Yellow indicates lactone. Green indicates the oxygen of diaryl ethers listed in the Table in (**b**).

Hatano also isolated alnusnin A (**148**) and platycaryanin A (**147**) in 2012 [102]. In the NMR studies, they realized that: (1) the chemical shifts of H-3′ in the “depside bond”-forming lactonized (namely, depsidone) tergalloyl groups indicated more downfield shifts than those in the normal tergalloyl groups (Figure 52a); and (2) that the chemical shifts of H-6″ in the tergalloyl groups shift to be more upfield than those in the valoneoyl groups (Figure 52b). The “depside” refers to two aromatic moieties connected through an ester bond. These subtle but remarkable characteristics would be useful for structural determinations of other ellagitannins.

#### 3.5.4. Nobotanin B, C, E, G, H, I, J, and K

**Figure 53:**
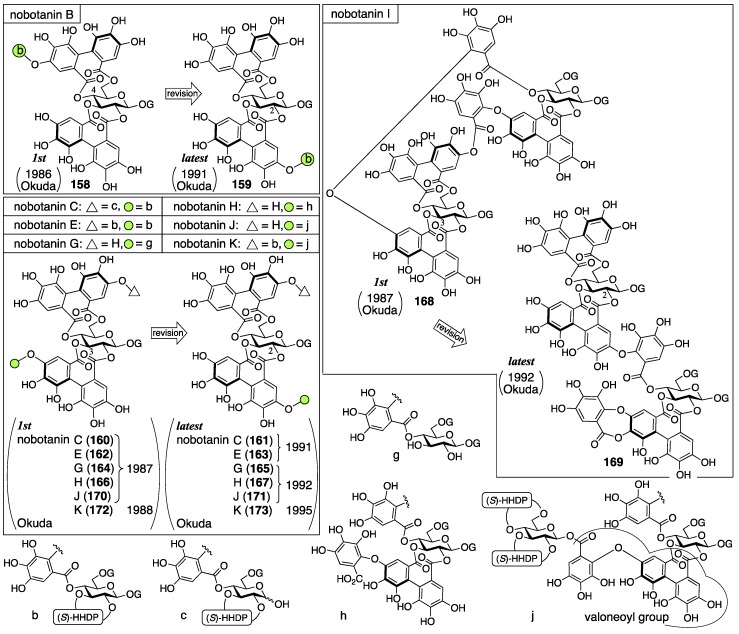
The transition of the structure of nobotanins B, C, E, G, H, I, J, and K. The green is merely a mark. The usage is the same in the following figures.

Nobotanins are ellagitannins derived from *Tibouchina semidecandra* and other plants and involve 22 analogs: nobotanin A–V. Among them, the initial structures of B, C, E, and G–K have been revised (Figure 53). For the nobotanins, the isolations, structural determinations, and revisions were all conducted by Okuda. The first structure of nobotanin B (**158**) was provided in 1986 [103]. In the following year, the first structures of nobotanins C (**160**), E (**162**), G (**164**), H (**166**), I (**168**), and J (**170**) were determined [104]. In 1988, the tetrameric structure **172** was reported for nobotanin K [105]. The main point of the changes in the structural revisions was the direction of the valoneoyl group. Firstly, the structures of nobotanin B, C, and E were corrected to **159**, **161**, and **163**, respectively, in 1991 [106]. In the ensuing year, the latest structures were reported for nobotanin G (**165**), H (**167**), I (**169**), and J (**171**) [107]. The revision of nobotanin I involves the change of the direction of the valoneoyl group and of its lactone structure from the macrocyclic ring to the seven-membered one. In 1995, nobotanin K was corrected to be **173** [108].

**Figure 54:**
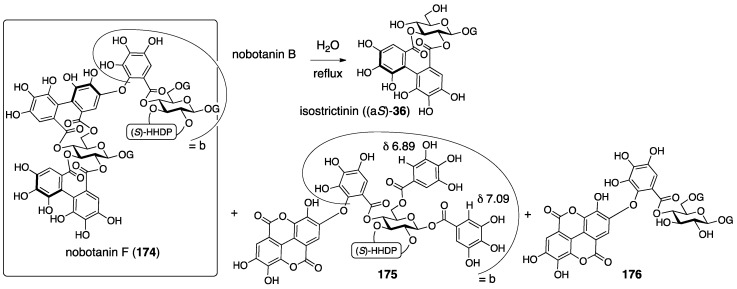
The information that led to the first structure of nobotanin B (**158**).

Among the nobotanins, initially, the first structure of nobotanin B (**158**) was determined along with nobotanin A, D, and F [103]. The CD and ^1^H/^13^C-NMR spectra indicated that nobotanin B was an isomer of nobotanin F (**174**) (Figure 54). In the hydrolysis of nobotanin B, the 1:1 production of isostrictinin ((a*S*)-**36**) [109] and **175** was observed at an early stage. The prolonged reaction time increased the yield of **176**. The structures for **175** and **176** were provided through a subsequent process. Thus, the ^1^H-NMR and UV spectra of **175** and **176** indicated the existence of: (1) the galloyl group; and (2) the valoneoyl group, in which the HHDP part was lactonized to be the structure of ellagic acid. The reason for the determination, which was that one of the two galloyl groups was on O-1, was the observation that the tannase treatment of **175** provided a hydrolysate that reacted at O-1. The position of the HHDP group in **175**, which was between O-2 and O-3, came from comparison of the ^1^H-NMR spectra of **175** and **176**. Regarding the remaining O-4 and O-6, on which the other galloyl group and the lactonized valoneoyl group were situated, their positions were determined on the basis of an unusual upfield shift (δ 6.89) of a hydrogen on one galloyl group. Okuda considered that the shift was the result of the magnetic anisotropy effect derived by the ellagic acid moiety. Because the shift was observed on only one of the galloyl groups, the authors distributed the second galloyl group onto O-6 and the lactonized valoneoyl onto O-4. Structure **158** was the combination of (a*S*)-**36** and **175**, with the exception of **174**.

**Figure 55:**
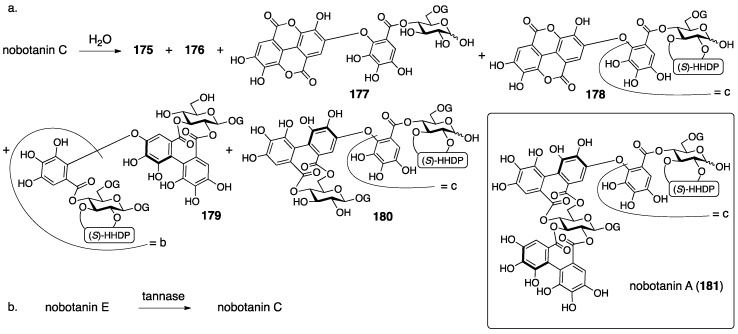
The information referred to in the structural determination of the first structures of nobotanins C (**160**) and E (**162**).

The first structures of nobotanins C (**160**) and E (**162**) were introduced on the basis of their hydrolysis [104]. The grounds for nobotanin C being a trimeric ellagitannin was its ^1^H/^13^C-NMR spectra and a GPC analysis. The ^1^H-NMR indicated the existence of two valoneoyl, two HHDP, and four galloyl groups, each as a 5:3 pair of signals. Therefore, nobotanin C was assessed as a mixture of anomers. Hydrothermal degradation provided **175**, **176**, **177**, **178**, **179**, and **180** (Figure 55a). Among them, **175**, **176**, **177**, and **178** were the known degradation products of nobotanins B and F (**174**) [103]; **178** was identical to the degradation product of nobotanin A (**181**) [103]; and **179** was that which was derived from nobotanin B. Note that structure **179** should be corrected according to the structural revision of nobotanin B to the latest structure. The structure of **180** was presumed based on its ^1^H-NMR spectrum. The *S*-axial chiralities for all the HHDP groups of nobotanin C were determined on the basis of the CD spectrum. Although arranging the above information could not narrow the direction of the valoneoyl group to one, structure **160** was illustrated according to the structure of nobotanin I, the structural determination of which is explained in the following paragraph. The reason that structure **162** was given for nobotanin E, which was the galloylated nobotanin C, was that the tannase hydrolysate of nobotanin E was nobotanin C (Figure 55b).

**Figure 56:**
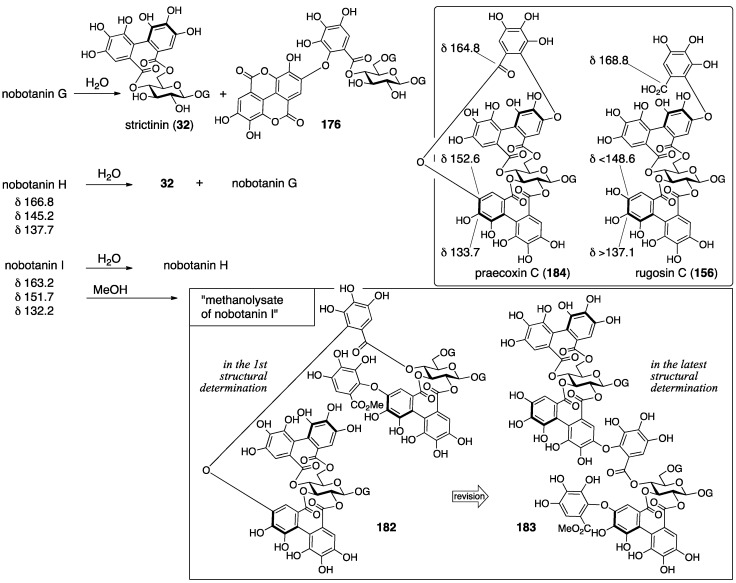
The observations that led to the first structures of nobotanins G, H, and I (**164**, **166**, and **168**).

The first structures of nobotanins G (**164**), H (**166**), and I (**168**) were revealed by association of their hydrolysates to related known compounds [104]. The ^1^H-NMR and COSY spectra led to the dimeric structure for nobotanin G, in which the O-2 and O-3 of one of the two glucoses were hydroxy groups and the other hydroxy groups were all acylated. With the observation that strictinin (**32**) and **176** were given as the hydrolysates (Figure 56) [103], structure **164** was provided. The reason for the determined direction of the valoneoyl group is described later. Nobotanin H was presumed to have the structure that a valoneoyl group was attached to nobotanin G on the basis of the ^1^H-NMR and COSY spectra. The additional information that the hydrolysis of nobotanin H produced **32** and nobotanin G led to structure **166**. Leaving a 0.1% aqueous solution of nobotanin I at 37 °C for 36 h provided nobotanin H quantitatively. In addition, leaving a solution of nobotanin I in MeOH at room temperature for a week provided a methanolysate of nobotanin I (**182**) quantitatively. Such hydrolysis and methanolysis that proceeded under mild conditions had been observed on compounds containing depside bonds (Section 3.5.3 for the meaning of depside). Therefore, the results strongly supposed the existence of a depside bond in nobotanin I. The comparison of the ^13^C-NMR spectra of nobotanin H to nobotanin I demonstrated that the signals at δ 166.8 and 137.7 of nobotanin H were shifted to a higher magnetic field to be δ 163.2 and 132.2, respectively. By contrast, the signal δ 145.2 of nobotanin H shifted lower to be δ 151.7 in nobotanin I. This phenomenon had been observed in the comparison of rugosin C (**156**) and praecoxin C (**184**) [110]. Accordingly, the structure of nobotanin I was considered to have a lactonized structure of nobotanin H, where the direction of the valoneoyl group was attributed by considering a molecular model in which the diaryl ether part was situated on the O-3 side of the glucose. Hence, the structure was **168** (Figure 53). The determined direction of the valoneoyl group affected the structure of nobotanins C (**160**), E (**162**), G (**164**), and H (**166**).

**Figure 57:**
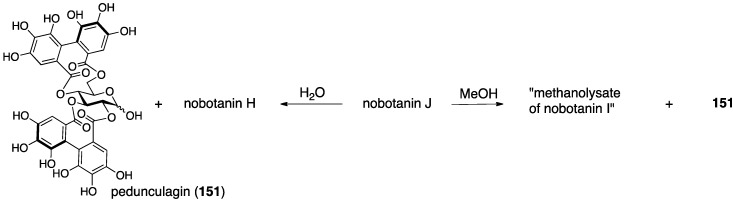
The transformation used in the structural determination for the first structure of nobotanin J (**170**).

The first structure of nobotanin J (**170**) was determined according to its fragments [104]. The ^1^H/^13^C-NMR spectra indicated: (1) that the compound was trimeric; (2) the existence of two valoneoyl, three HHDP, and three galloyl groups; and (3) that all the hydroxy groups of glucose were acylated. A dilute aqueous solution of nobotanin J hydrolyzed it to provide pedunculagin (**151**) and nobotanin H (Figure 57). Leaving its methanol solution at room temperature gave a compound that was the same as the “methanolysate of nobotanin I” and **151**.

For the structural determination of nobotanin K (**172**), nothing has been reported to international journals. A summary of a conference presentation suggests that the structure was identified on the basis of a comparison of the decomposed product of nobotanin K to nobotanins E and J [105].

**Figure 58:**
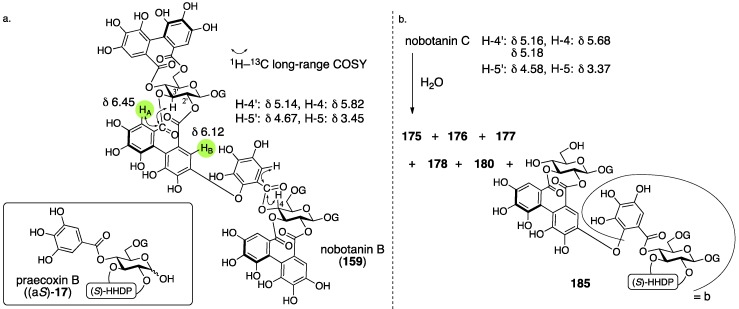
The data used for the revision to the latest structures of nobotanins B, C, and E (**159**, **161**, and **163**, respectively).

Okuda revised nobotanins B, C, and E to their latest structures, **159**, **161**, and **163**, respectively, in 1991 [106]. In the structural revision of nobotanin B, the position of the valoneoyl group was changed. It had been known that the NMR chemical shifts of hydrogens on the HHDP moiety of a valoneoyl group indicated an obvious difference at the sides with and without the aryl-O-aryl bond [111,112,113]. According to this knowledge, the hydrogens H_A_ and H_B_ of the valoneoyl groups were assigned as illustrated in Figure 58a. Among them, the H_A_ (δ 6.45) was associated with the H-3′ of glucose in a ^1^H–^13^C long-range COSY experiment, which determined the direction of the valoneoyl group. In addition, the long-range data ensured that the galloyl moiety of the valoneoyl group was attached to the O-4 of glucose, the bonding position of which had remained uncertain since the structural determination in 1986. Note that the clear differences in the chemical shifts—δ H-4′ < δ H-4 and δ H-5′ > δ H-5—were observed in the ^1^H-NMR spectrum of nobotanin B. This phenomenon is a common feature when the HHDP part of a valoneoyl group is attached to the O-2′ and O-3′ of one glucose and the galloyl part is connected to the O-4 of the other glucose as seen in **159** [113]. In the revision to the latest structure of nobotanin C (**161**), the following three facts served as the evidence. First, the differences in the chemical shifts between H-4′ and H-4 and between H-5′ and H-5 were similar to those in nobotanin B (Figure 58b). Second, in the ^1^H/^13^C-NMR spectrum, the signals due to glucose moieties resembled those of nobotanin B and praecoxin B ((a*S*)-**17**) [110] closely. Third, compound **185** was found among the hydrolysates of nobotanin C. The structure of **185** was revealed using ^1^H-NMR and mass spectra. Nobotanin E had been known as galloylated nobotanin C (Figure 55b); therefore, the structure was revised to be **163**.

**Figure 59:**
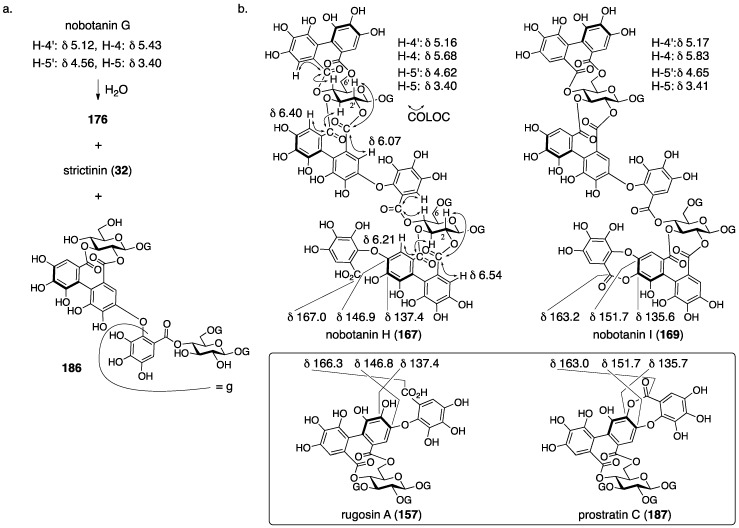
The information that led to the latest structures of nobotanins G, H and I (**165**, **167** and **169**, respectively).

The common point in the revision to the latest structures of nobotanins G–J is the overturning of the valoneoyl group [107,114]. The structural revision of nobotanin G was ascribed to the observations that: (1) the differences in the chemical shifts between H-4′ and H-4 and between H-5′ and H-5 were similar to those in nobotanin B; and (2) that **186** was found among the hydrolysates of nobotanin G (Figure 59a). The grounds for the structural revision of nobotanin H were: (1) the similar differences in the chemical shifts between H-4′ and H-4 and between H-5′ and H-5; and (2) the ^1^H–^13^C long-range shift correlation coherence spectroscopy (COLOC) (see **167** in Figure 59b). The bases for the structural revision of nobotanin I were: (1) the revision of nobotanin H; and (2) the comparison of the ^13^C-NMR chemical shifts of nobotanin H to rugosin A (**157**) [112] and prostratin C (**187**) [115,116]. Regarding the assertion (1), as nobotanin H was a hydrolysate of nobotanin I (Figure 56), the structural revision of nobotanin H affected the structure of nobotanin I. In terms of the assertion (2), the feature of the ^13^C-NMR chemical shifts of the carbons that might construct the seven-membered lactone observed in the non-lactonized **157** and the lactonized **187** were exactly like those observed in nobotanins H and I [115]. The structural revision of nobotanin J came from the revision of nobotanin H.

**Figure 60:**
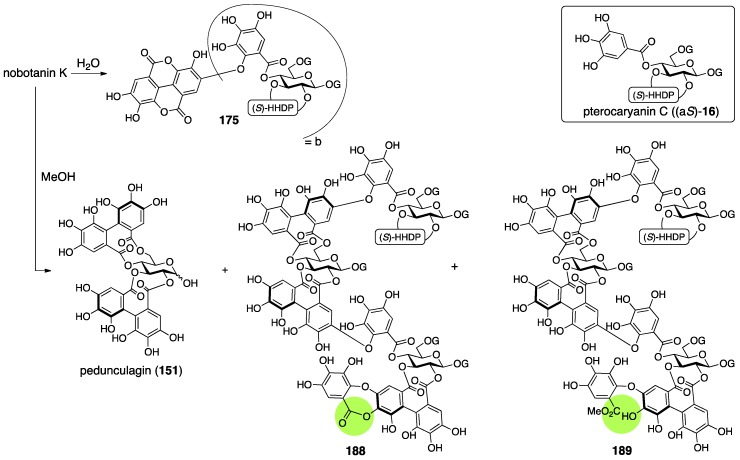
The information used for the revision to the latest structure of nobotanin K (**173**).

The structure of nobotanin K was revised in 1995 [108]. On the occasion of the revision, the tetrameric structure and existence of three valoneoyl, three HHDP, and five galloyl groups were reconfirmed. Another reaffirmation was that nobotanin K had a structure that was composed of nobotanin J and pterocaryanin C ((a*S*)-**16**), which was carried out by a comparison of the NMR data of it to nobotanin J and (a*S*)-**16** [114]. Therefore, the structure of nobotanin K was influenced by the revision of nobotanin J. The hydrothermal degradation of nobotanin K provided **175** (Figure 60), which had also been obtained by the hydrolysis of nobotanins B, E, and F (**174**). On the other hand, the methanolysis of nobotanin K gave **188** and **189** in addition to **151**. The structures of **188** and **189** were determined on the basis of their ^1^H-NMR spectra. Taking the above information together, the structure was revised to **173**.

## 4. Summary

Table 1 summarizes the causes for wrong structures, how the error was realized, and the methods used for latest structural revision for each ellagitannin introduced in this review. The content given to each item is expressed roughly since the principal description has already been given in Section 3. The most common cause for a wrong structure is in a prediction based on “similarity” (red text), where CD/ORD is the most-used data for the similarity.

The most common reason for noticing structural errors is a structure determination on similar compounds (green text). A contradiction between NMR data and the reported structure is also a common reason (light blue text). Indication of errors in ellagitannin structures by total synthesis is a minor reason.

In the latest structural determination, methods based on identification are chiefly used (blue text). The reason why there are only two examples using X-ray analysis is probably due to the unique circumstances of ellagitannins, for which it is difficult to obtain good single crystals. The long-range correlation methods in NMR spectroscopy, such as the HMBC method, which had been generalized in the 1990s, is quite useful for a structural determination on ellagitannins (purple text). Structure determination incorporating support for chemical calculation is a new trend, and this method revised the structures of castalin, vescalin, castalagin, and vescalagin 25 years after the previous structure determination (Section 3.4.1). These are revisions of those structures that had previously been “believed”. By the way, among the ellagitannins appearing in this review, the initial structure of roxbin B ((a*S*,a*S*)-**27**) had been believed for 27 years, which is the longest period. However, roxbin B disappeared as the compound was found to have the same structure as an already known ellagitannin, cuspinin ((a*R*,a*S*)-**2**) (Section 3.1.4).

Recently, support for computational chemistry in structure determination [117] and techniques for an X-ray diffraction analysis without crystallization are advancing [118]. As examples that have already been used in the structural revision of ellagitannins, these new methods might change the isolation/structural determination protocols, together with the advancement of separation methods, to enable the use of smaller amounts of samples with a shorter time period. In the course of promoting the new methods, verification using “identification” based on reliable information will be important.

## Figures and Tables

**Table 1:** Causes for wrong structures, how the error was realized, and methods used for the latest structural confirmation.

Section	Compound Name	Cause(s) for Wrong Structure	How the Error Was Realized	Methods Used for Latest Structural Confirmation
4.1.1.	corilagin	Prediction based on similarity of CD/ORD spectra	unclear	Identification with a known fragment
				Total synthesis and identification
4.1.2.	punigluconin	Misassignment of NMR data	Structural determination of analogous compounds	NMR studies
4.1.3.	cercidinins A and B	Prediction based on similarity of NMR data with analogous compounds	Total synthesis	NMR studies with long-range methods
				Total synthesis and identification
4.1.4.	roxbin B	Prediction based on similarity of CD/ORD spectra	Total synthesis	Identification of NMR data to a known compound
				Total synthesis and identification
4.2.1	geraniin	Misinterpretation of NMR data	Contradiction between NMR data and reported structure	NMR studies
				Single-crystal X-ray diffraction
4.2.2.	terchebin	Prediction based on analogous compounds Incorrect experimental results	Contradiction between NMR data and reported structure	Structural determination from the beginning
4.2.3.	isoterchebin	Incorrect experimental results	Identification of the reported structure to the other compound	Structural determination from the beginning involving identification
4.3.	chebulinic acid and chebulagic acid	Unreasonable structure determination under lack of evidence	Contradiction between NMR data and reported structure	Structural determination from the beginning
				Single-crystal X-ray diffraction of a fragment
4.4.1.	castalin, vescalin, castalagin, vescalagin, casuarinin, and stachyurin	Misinterpretation of NMR data	unclear	NMR studies
		Prediction based on similarity of CD/ORD spectra		Chemical calculation
				Total synthesis and identification
4.4.2.	punicalin and punicalagin	Use of molecular model for the final basis	Structural determination of analogous compounds	Structural determination from the beginning involving identification
4.5.1	sanguiin H-2, H-3, and H-6	Prediction based on similarity of CD/ORD spectra	Structural determination of analogous compounds	Structural determination from the beginning
				Synthesis of a fragment and identification
4.5.2.	alnusiin	Use of the additivity of the substituent effect in the ^13^C-NMR spectrum	unclear	NMR studies with long-range methods
4.5.3	alnusnins A and B	Incorrect experimental results	Structural determination of analogous compounds	Correct mass spectra
4.5.4.	nobotanins B, C, E, G, H, I, J, and K	Prediction based on wrong structures	Structural determination of analogous compounds	NMR studies with long-range methods
		Use of molecular model for the final basis		

Red: prediction based on “similarity”. Green: structural determination on similar compounds. Lite blue: contradiction between NMR data and the reported structure. Blue: methods based on identification. Purple: use of long-range correlation methods in NMR spectroscopy. CD, circular dichroism; ORD, optical rotatory dispersion.

## Abbreviations

Ac	acetyl
All	allyl
aq	aqueous
Bn	benzyl
CD	circular dichroism
COLOC	correlation spectroscopy via long-range coupling spectrum
conc	concentrated
COSY	correlation spectroscopy
D	deuterium, dextrorotatory
DCC	*N*,*N*′-dicyclohexylcarbodiimide
DFT	density functional theory
DHHDP	dehydrohexahydroxydiphenoyl
DIS	differential isotope shift
DMAP	4-(dimethylamino)pyridine
DMF	*N*,*N*-dimethylformamide
DMSO	dimethyl sulfoxide
ECD	electronic circular dichroism
EDCI·HCl	1-(3-dimethylaminopropyl)-3-ethylcarbodiimide hydrochloride
Et	ethyl
*epi*	epimer
FAB-MS	fast atom bombardment-mass spectrometry
G	galloyl
G(Ac_3_)	tri-*O*-acetylgalloyl
G(Bn_3_)	tri-*O*-benzylgalloyl
G(All_2_Bn)	3,5-di-*O*-allyl-4-*O*-benzylgalloyl
G(Me_3_)	tri-*O*-methylgalloyl
GPC	gel permeation chromatography
h	hour(s)
HHDP	hexahydroxydiphenoyl
HHDP(Me_6_)	hexa-*O*-methyl-hexahydroxydiphenoyl
HMBC	hetero-nuclear multiple-bond coherence
*i*	iso
IR	infrared
Me	methyl
MP	4-methoxyphenyl
MS	mass spectrum (or spectra)
MS 4A	molecular sieves 4A
*n*	normal
NHTP	nonahydroxytriphenoyl
NMR	nuclear magnetic resonance
NOE	nuclear Overhauser effect
ORD	optical rotatory dispersion
Ph	phenyl
Pr	propyl
*rac*	racemic
rt	room temperature
*t*	tertiary
TBAF	tetra-*n*-butylammonium fluoride
TBS	*t*-butyldimethylsilyl
THF	tetrahydrofuran
UV	ultraviolet
